# Mammal extinction facilitated biome shift and human population change during the last glacial termination in East-Central Europe

**DOI:** 10.1038/s41598-022-10714-x

**Published:** 2022-04-26

**Authors:** Enikő Katalin Magyari, Mihály Gasparik, István Major, György Lengyel, Ilona Pál, Attila Virág, János Korponai, Aritina Haliuc, Zoltán Szabó, Piroska Pazonyi

**Affiliations:** 1 MTA-MTM-ELTE Research Group for Palaeontology, Budapest, Ludovika tér 2, 1083 Hungary; 2 Department of Environmental and Landscape Geography, Eötvös Loránd University, Budapest, Pázmány Péter stny 1/c, 1117 Hungary; 3 Isotope Climatology and Environmental Research Centre (ICER), Institute for Nuclear Research, Hungarian Academy of Science, Debrecen, Bem tér 18/c, 4026 Hungary; 4 Department of Palaeontology and Geology, Hungarian Natural History Museum, Budapest, Ludovika tér 2, 1083 Hungary; 5 Department of Prehistory and Archaeology, University of Miskolc, Miskolc-Egyetemváros, 3515 Hungary; 6 Department of Mineralogy and Geology, University of Debrecen, Debrecen, Egyetem tér 1, 4032 Hungary; 7 Department of Water Supply and Sewerage, University of Public Service, Baja, Bajcsy-Zsilinszky utca 12-14, 6500 Hungary; 8 Department of Environmental Sciences, Sapientia Hungarian University of Transylvania, Calea Turzii str. 4, 400193 Cluj-Napoca, Romania; 9 Department of Danube’s Diversity, Institute of Aquatic Ecology, Centre for Ecological Research, Budapest, Karolina út 29, 1113 Hungary; 10 Institute of Speleology, Romanian Academy, 5 Clinicilor str, 400006 Cluj-Napoca, Romania; 11 EPOC, UMR 5805, Université de Bordeaux, Allée Geoffroy St Hilaire Bat B18N, CS 50023, 33615 Pessac Cedex, France

**Keywords:** Palaeontology, Biogeography, Palaeoecology

## Abstract

The study of local extinction times, together with the associated environmental and human population changes in the last glacial termination, provides insights into the causes of mega- and microfauna extinctions. In East-Central (EC) Europe, groups of Palaeolithic humans were present throughout the last glacial maximum, but disappeared suddenly around 15,200 cal BP. In this study cave sediment profiles dated using radiocarbon techniques and a large set of mammal bones dated directly by AMS ^14^C were used to determine local extinction times. These were, in turn, compared to changes in the total megafauna population of EC Europe derived from coprophilous fungi, the Epigravettian population decline, quantitative climate models, pollen and plant macrofossil inferred climate, as well as to biome reconstructions. The results suggest that the population size of large herbivores decreased in the area after 17,700 cal BP, when temperate tree abundance and warm continental steppe cover both increased in the lowlands. Boreal forest expansion started around 16,200 cal BP. Cave sediments show the decline of narrow-headed vole and arctic lemming populations specifically associated with a tundra environment at the same time and the expansion of the common vole, an inhabitant of steppes. The last dated appearance of arctic lemming was at ~ 16,640 cal BP, while that of the narrow-headed vole at ~ 13,340, and the estimated extinction time of woolly mammoth was either at 13,830 (GRIWM) or 15,210 (PHASE), and reindeer at 11,860 (GRIWM) or 12,550 cal BP (PHASE). The population decline of the large herbivore fauna slightly preceded changes in terrestrial vegetation, and likely facilitated it via a reduction in the intensity of grazing and the concomitant accumulation of plant biomass. Furthermore, it is possible to conclude that the Late Epigravettian population had high degree of quarry-fidelity; they left the basin when these mammals vanished.

## Introduction

Despite the richness of vertebrate records from cave deposits and open air sites from the Late Pleistocene^1^, twentieth century paleontological research did not take the opportunity to date rigorously the latest appearance of large mammals and rodents during the last glacial termination in EC Europe (the eastern part of Central Europe). It also failed to date systematically cave mammal bone stratigraphies for the last 40,000 years, a time period for which AMS ^14^C dating would have been a perfect choice^2,3^. Only in recent years has a resurgence of Upper Weichselian megafauna research been seen, with researchers revisiting key localities, often associated with human occupation, and reassessing their dating^4–6^. As our understanding of Late Epigravettian human lifestyle, economy and hunting strategy increases, the clearer it becomes that the disappearance of mobile Epigravettian groups from the Carpathian Basin (CB) around 15,200 cal BP is related to changing environmental conditions, such as the rapid disappearance of their main quarry, the reindeer (*Rangifer tarandus*), the decimation of secondary quarries such as the wild horse (*Equus ferus*), not to mention the worsening visibility for hunting in a landscape subject to an increase in forest cover^7,8^.

Here, the focus is on one species of megafauna, the woolly mammoth (*Mammuthus primigenius*), and several ungulate and rodent species that co-inhabited the CB in a fully developed glacial ecological setting, and what is more, one that is relatively well understood^9–14^.

**Figure 1:**
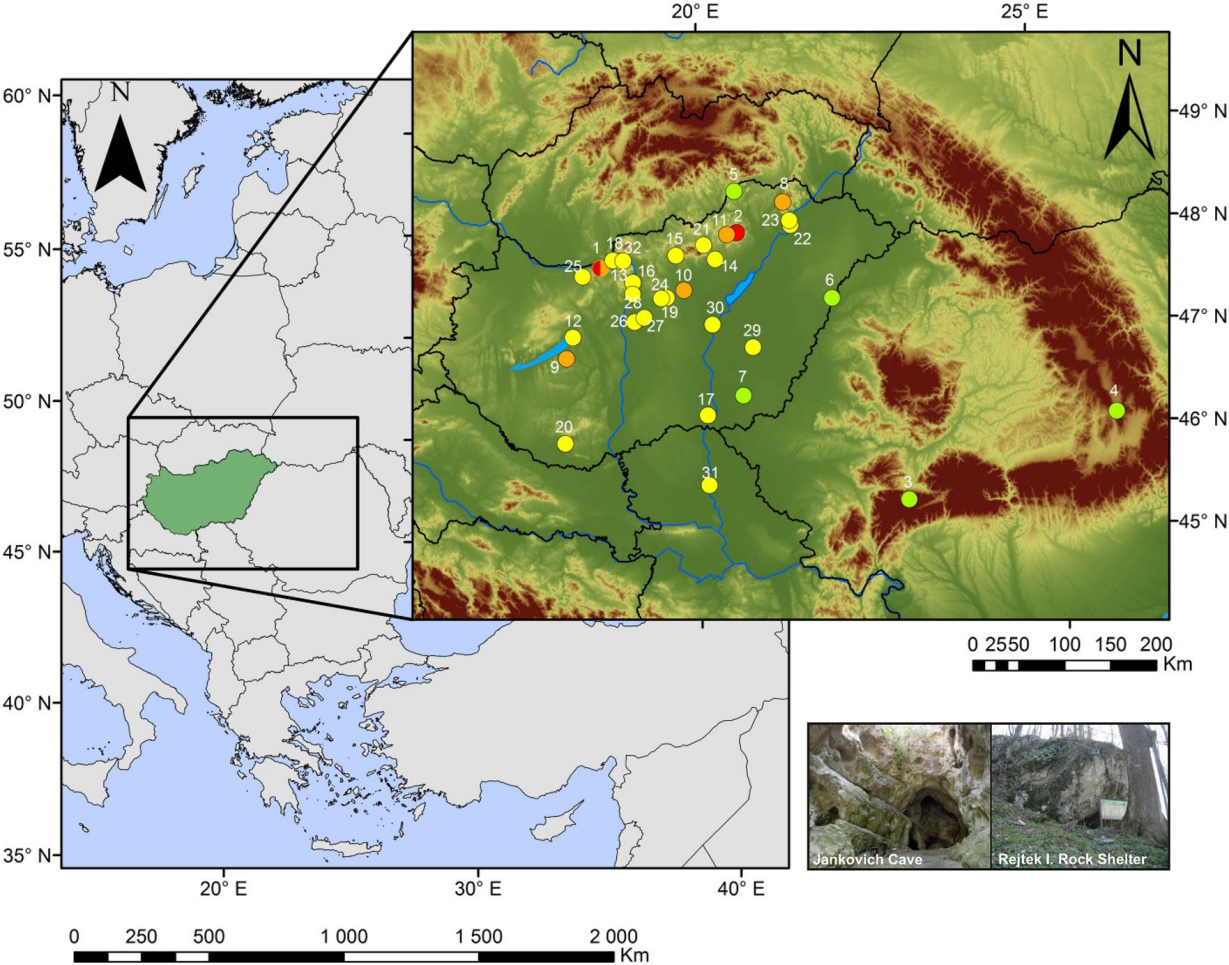
Location of the studied cave sites (red, 1–2), pollen records (green, 3–7), dated reindeer (*Rangifer tarandus*) (orange, 1, 8–11) and dated woolly mammoth (*Mammuthus primigenius*) bones (yellow, 12-32) in the Carpathian Basin (**a**) and in East-Central Europe (**b**). 1: Jankovich Cave; 2: Rejtek I Rock Shelter.; 3: Taul dintre Brazi^16^; 4: Lake St Anne^13^; 5: Kelemér Nagymohos^17^; 6: Kokad Mire^18^; 7: Kardoskút Fehér Lake^19^; 8: Arka; 9: Ságvár; 10: Jászfelsőszentgyörgy ; 11: Peskő Cave; 12: Csajág; 13: Pilismarót; 14: Feldebrő; 15: Szurdokpüspöki; 16: Budapest-Csillaghegy; 17: Szeged-Öthalom; 18: Esztergom-Gyurgyalag; 19: Tápiósüly; 20: Zók; 21: Mátraderecske; 22: Tarcal; 23: Bodrogkeresztúr; 24: Mende; 25: Tata-Porhanyó quarry; 26: Kiskunlacháza; 27: Ócsa-Felsőbabád; 28: Lágymányos; 29: Gyoma, River Tisza; 30: River Tisza; 31: Törökbecse; 32: Zebegény. The software used to create the maps was ArcGIS 10.2.2 for desktop, version 10.2.2.3552. Software url: https://support.esri.com/en/products/desktop/arcgis-desktop/arcmap/10-2-2.; the photo of Rejtek I Rock Shelter was taken by Tivadar Czina under licence CC BY-SA 3.0, *source*: https://hu.wikipedia.org/wiki/Rejteki_1._sz._kőfülke; the photo of Jankovich Cave was taken by Mihály Gasparik.

Using a large dataset comprising dated mammoth bones, two profiles of caves with known human occupation and five multi-proxy paleoecological records from lake and mire sediments covering the last glacial maximum (LGM) and last glacial termination (Fig. 1), a specific paleoecological hypothesis relating rapid climate change to population dynamics is put to the test, namely, that transitions from cold to warm intervals were briefly optimal for grazing megafauna, but these brief optima were followed by rapid regional extinctions^15^. The question of the order of faunistic and vegetation biome changes and its casual linkage is also examined.

### Studied cave sites and the mammoth bone collections

The caves under consideration are in Northern Hungary, in the Pre-Carpathian hill region (Fig. 1). The Jankovich Cave is an archaeological site in the Gerecse Hills. In the present study the Late Upper Palaeolithic layer is in focus^20,21^ that likely belongs to the Late Epigravettian. Situated at 330 m a.s.l., its 88 m long chamber has been excavated repeatedly since 1913; Block II, excavated by László Vértes in 1956, was selected for examination (Suppl. Fig. 1). This revealed that microfauna adapted to a cold climate were replaced by temperate species^21^, and was therefore assigned to the Pleistocene/Holocene boundary. 21 bone samples from 13 layers were selected for AMS ^14^C dating (Suppl. Tables 1 and 2).

The Rejtek I Rock Shelter is situated in the Bükk Mts at 534 m a.s.l. (Fig. 1, Suppl. Fig. 2). Here, the AMS ^14^C measurements were carried out on Profiles II (upper 4 layers) and III (layers 5–8)^22^. These two profiles had earlier been combined on the basis of depth^23^, and faunistic and floristic changes were published accordingly (Suppl. Figs. 2 and 3; Suppl. Tables 1 and 3). Knapped lithics were found^24^, charcoal, mollusc and bone assemblages were analysed^4,25,26^. 24 bone samples from 8 layers were selected for dating (Suppl. Tables 1 and 3). Priority was given to mammal species with missing or uncertain data on their last occurrence: e.g. steppe pika (*Ochotona pusilla*), arctic lemming (*Dicrostonyx torquatus*), narrow-headed vole (*Lasiopodomys* (*S*.) *gregalis*), reindeer (*R*. *tarandus*), and willow grouse (*Lagopus lagopus*).

The woolly mammoth was common in the CB during the Weichselian glaciation; *ca*. 400 specimens and 6 skeletons had been found in Hungary, but ^14^C dates were only available for 8 specimens^27^. Within the scope of this study, 21 localities (Suppl. Table 4, Fig. 1) were selected for AMS ^14^C dating.

The cave localities, lake and mire sites used for the pollen-based climate and biome reconstructions in this study are described in detail in the Supplementary Material.

## Results

### Last glacial termination faunal changes and last detection times

Stratigraphically consistent dates were obtained in both caves below a depth of 1 m of sediment (layers 8–12 in Rejtek, layers 5–10 in Jankovich), while in the top meter, mixed Holocene and Pleistocene ages indicated significant disturbance (Suppl. Tables 1, 2 and 3). The age range of the bone assemblages in the two caves is different: Jankovich layers 5–10 date between 17,550 and 15,300 cal BP, while the Rejtek layers 8–12 span 13,450–9950 cal BP. The Pleistocene-Holocene transition could only be traced at Rejtek. The age-depth models (Suppl. Fig. 4) suggest that sediment accumulation rates were relatively even in both caves, *ca*. 0.5 mm yr^−1^.

Major vole faunal changes in the Jankovich profile suggest that the common vole (*Microtus arvalis*), narrow-headed vole (*L*. (*S*.) *gregalis*) and arctic lemming (*D. torquatus*) were dominant after the last glacial maximum (LGM: 26–21 ka cal BP) up until 16,360 cal BP. This assemblage was replaced by common vole (*M*. *arvalis*) and bank vole (*Clethrionomys glareolus*), which achieved dominance by 15,540 cal BP, suggesting significant warming and the disappearance of steppe-tundra habitats.

**Figure 2:**
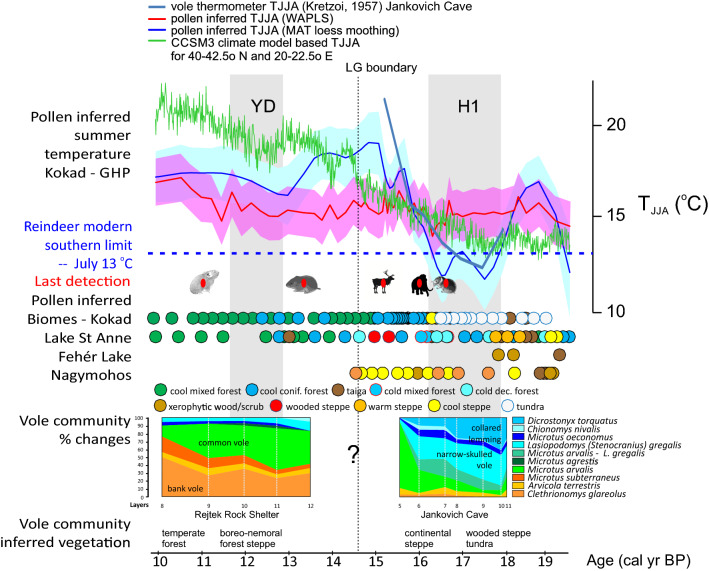
Vole community changes through time in two Carpathian Basin cave sediment profiles AMS ^14^C dated within the scope of this study (Jankovich Cave, Rejtek Rock Shelter); vegetation inferred from the vole community; reconstruction of the biome on the basis of five pollen records from the Carpathian Region (for locations see Fig. 1); last detection times of micro- and macrofauna elements in this study (from left: *Ochotona pusilla*, *Lasiopodomys* (*S*.) *gregalis*, *Rangifer tarandus*, *Mammuthus primigenius*, *Dicrostonyx torquatus*), pollen inferred summer mean temperature reconstructions (WAPLS, MAT) on the basis of the Kokad Mire pollen records (redrawn and modified from Magyari et al.^18^) and CCSM3 climate model based summer mean temperature simulation for the Kokad grid cell from PaleoView; animal drawings on this figure were made in Corel Draw version 23.0.0.363 by the first author.

Radiocarbon dating confirms that this change started after Heinrich event 1 (HE-1) and had ended by the onset of the Bølling/Allerød Interstadial (GI-1e: ~ 14,700 cal BP) (Fig. 2; Suppl. Fig. 5). The onset of the Holocene is characterised by an increase in the forest dwelling bank vole (*C. glareolus*). A transition from wooded steppe tundra to continental steppe may be inferred as taking place between 15,540 and 16,000 cal BP (Fig. 2). The Jankovich vole record implies that the dominance of the arctic lemming and tundra vole (*D. torquatus* & *M. oeconomus*) between 17,000 and 16,300 cal BP may well be connected to the HE-1 cooling (Suppl. Fig. 5). The steppe pika (*O*. *pusilla*) retreated after this interval (Suppl. Table 1).

The large mammalian fauna of Jankovich also indicates this climatic change: the cave bear (*Ursus spelaeus*), reindeer (*R*. *tarandus*), cave lion (*Panthera* (*Leo*) *spealea*), arctic fox (*Vulpes lagopus*) and horse (*Equus sp*.) that characterize the lower layers gradually disappear upwards in the stratigraphy. It should, however, be noted that the large mammalian bones were not directly dated using ^14^C.

Macrofauna, vole and rodent relative frequency from Rejtek I (Fig. 2; Suppl. Fig. 6) demonstrate that accumulation started in the Allerød (~ GI-1b) and a major change is the shift in dominance from the common vole to the bank vole (*M. arvalis* – > *C. glareolus*) at 12,200–11,700 cal BP. This correlates with the Holocene transition. The relative frequencies of the European pine vole (*M. subterraneus*) and wood mouse (*Apodemus sylvaticus*) also increase indicating warming and forest cover increase. In the Allerød warm period, willow grouse (*L*. *lagopus*; dated to 14,610–15,190 cal BP; Suppl. Table 1), rock ptarmigan (*L*. *mutus*), steppe pika (*O. pusilla*), reindeer (*R*. *tarandus*) and steppe bison (*Bison priscus*; bone with low collagen) were still present, but they disappear in the Holocene layers (Fig. 2; Suppl. Fig. 6). This suggests a Late Glacial (LG) persistence of some species adapted to cold taiga, forest tundra and steppe habitats.

This cave sequence is exceptional in terms of the detailed charcoal record present in the same sediment layers as those in which the mammal bones were found. As shown in Suppl. Fig. 3, the dominant trees in the LG landscape of Rejtek were needle leaved: the charred remains of Norway spruce (*Picea abies*) and Arolla pine (*Pinus cembra*) were found together with Scots pine (*P*. *sylvestris*). From *ca*. 13,000 cal BP onwards, a dominance of mixed deciduous, maple (*Acer platanoides*), field elm (*Ulmus campestris*), hornbeam (*Carpinus betulus*) and beech (*Fagus sylvatica*) suggested a LG expansion of several early and late successional deciduous trees^4,25^. Particularly striking was the appearance of beech and hornbeam. These spread later at 300 m a.s.l. in the Bükk Mts, around 6500 cal BP^28,29^, and heir LG presence supports isoensyme and chloroplast (cp) microsatellite DNA studies inferring possible cryptic populations in the N Hungarian Hills^30^. The mollusc fauna of Rejtek contained over 65% forest dwellers (Clausiliidae, *Cochlodina cerata*) in the LG and Early Holocene layers, indicating a forested landscape^4,31^.

**Figure 3:**
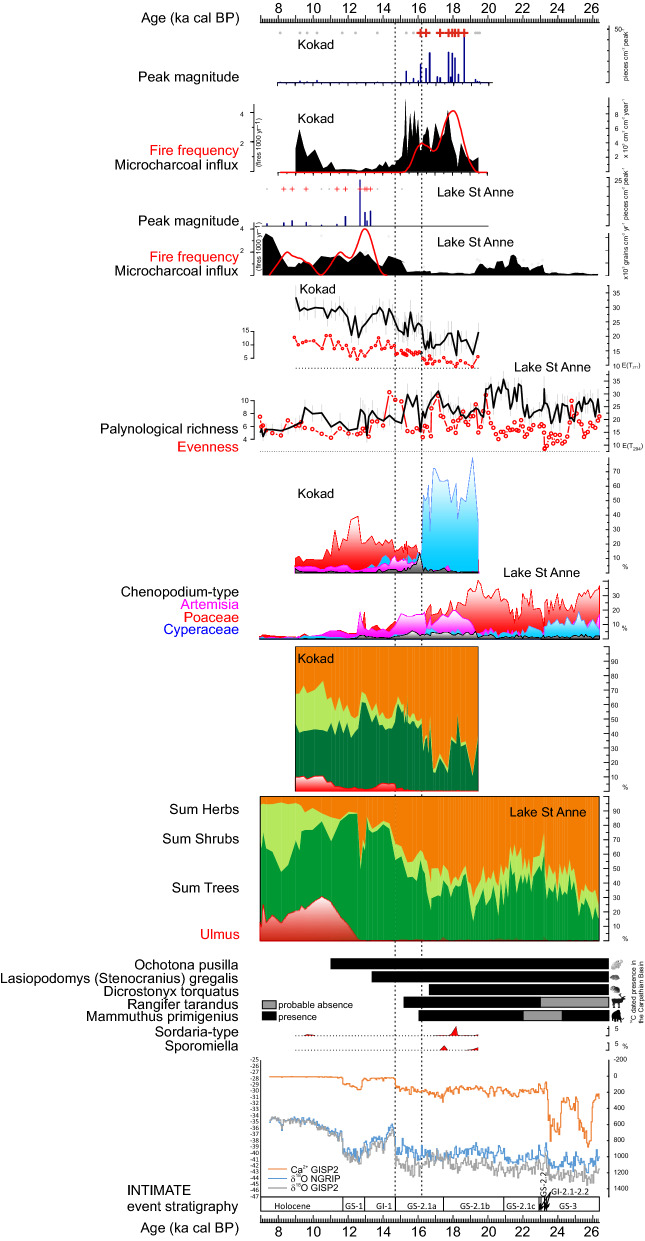
INTIMATE event stratigraphy plotted along the NGRIP and GISP2 δ^18^O and Ca^2+^ ice core records from Greenland^34^; selected dung fungal spores (*Sporomiella*, HdV55a) indicating the presence of large bodied grazers in the Kokad pollen profile^18^; calibrated BP intervals of presence for selected mega- and microfauna elements in the Carpathian Basin dated within the scope of this study; summary pollen diagrams from two sites (Lake St Anne—950 m a. s. l.^13^ and Kokad Mire—112 m a. s. l.^18^) showing the cumulative percentage of total tree, shrub and herb pollen types and elm (*Ulmus*) pollen, and separate non-cumulative relative frequency curves for major herb pollen types (*Chenopodium*-type, *Artemisia*, Poaceae, Cyperaceae), palynological richness, microcharcoal influx, macrocharcoal inferred fire frequency curves, peak fire magnitudes and fire events (red crosses) from the same pollen sites. The methodology of macrocharcoal analyses is described in the Supplementary Material.

^14^C dates suggest that the arctic lemming’s (*D*. *torquatus*) population significantly decreased in the Gerecse Hills around 16,640 cal BP, followed by the narrow-headed vole (*L*. (*S*.) *gregalis*) around 13,340 cal BP, while the steppe pika (*O. pusilla*) survived into the Early Holocene; the latest detection time is around 11,015 cal BP at Rejtek (Suppl. Table 1; Fig. 3). Several reindeer (*R. tarandus*) bones were also dated; the latest date was from a stratigraphically consistent, undisturbed layer: ~ 15,195 cal BP (Suppl. Table 5; Suppl. Fig. 7). Taking into account other published data on reindeer ^14^C dates in the CB (15 sites), the Phase model in OxCal^32^ and the GRIWM model^33^ were employed to estimate the last appearance of reindeer. The end boundary in the Phase model provides an estimate between 8300 and 14,480 cal BP, with a median at 12,550, while the GRIWM model places the extinction between 11,470 and 12,815 with an inverse weighted terminal age estimate of 11,860 cal BP (see Supplementary Material).

In the radiocarbon dating of the woolly mammoth (*M*. *primigenius*) bone assemblages from the CB (Suppl. Table 4; Suppl. Fig. 8), 12 of 40 bones had an insufficient collagen content, 16 samples were dated beyond 40,000 ^14^C BP, and 12 provided dates suitable for calibration.

Combined with previously published dates from the CB (Suppl. Fig. 8), the 2σ calibrated age ranges using Intcal20^35^ showed that the youngest woolly mammoth was from Csajág^36^, dated to 16,180 (15,830–16,010) cal BP. There are eight bones dated to < 20 ka, nine between 20 and 30 ka, and twenty beyond 30 ka cal BP. Although the number of dated bones is a fraction of the bones available in museum collections, an absence between 22–24.5 ka and 27.4–32.5 ka cal BP is apparent. These data further suggest that woolly mammoth was common in the CB after the LGM, until 18 ka cal BP, though population decline was strong by 16.2 ka cal BP, and the species likely persisted locally during the coldest LGM period. The Phase model (n = 26) suggests a last appearance between 12,690 and 16,150 cal BP, with a median at 15,210 cal BP. The GRIWM model narrows down the confidence interval, but also estimates a considerably younger extinction time (13,717–13,960, terminal age 13,830 cal BP). If the problematic Zók site and burnt bones are removed from the Phase modelling (n=22), the estimated last appearance interval is still very similar (12,050–16,110 cal BP; median: 15,270).

### Last glacial termination vegetation changes in pollen and plant macrofossil records

Pollen based biome reconstruction of four EC European localities (Fig. 2) suggests that the vegetation was diverse during and after the LGM, depending on elevation, water availability and soil type. Alluvial plains and mid elevation mountain sites were covered by cool coniferous forest steppes (e.g. around Fehér Lake and Lake St Anne), locally tundra vegetation was dominant in cold wet basins (Nagymohos Peat Bog, Kokad Mire), while xerophytic woodland and scrub and cold steppe biomes were also present (Fig. 2).

Biome shifts begin to prevail earliest between 17,000 and 16,000 cal BP. In the mountains, cold deciduous forest, cool coniferous forest and forest steppe replaced the dominant steppe and taiga biome of the preceding HE-1 cooling. A much more abrupt biome shift from tundra to cool coniferous forest took place at Kokad around 16,200 cal BP (Fig. 2)^18^; this was followed by a second biome shift to cool mixed forest at 14,740 cal BP, a biome that then persisted into the Early Holocene here. A similar biome shift towards mixed leaved (boreo-nemoral) forest was also detected in the Eastern Carpathian site at 14,500 cal BP, suggesting the expansion of temperate deciduous trees during the LG interstadial (GI-1).

At the lowland Kokad site, grazing indicator fungi (*Sporomiella*, Sordariales and *Sordaria*-type (HdV55a)) were present on pollen slides. The disappearance of these fungi took place at 16,780 cal BP (Fig. 3)^18^. Around the same time, the micro- and macro-charcoal accumulation rate increased in the sediment and stayed high until *ca*. 15,000 cal BP, suggesting recurrent wildfires in the region between 17,700 and 15,000 cal BP (Fig. 3.).

Comparing selected pollen types, palynological richness (representing floristic diversity here), evenness (a measure of abundance distribution within the pollen assemblages), microcharcoal accumulation rate (indicative of regional fires) and macrocharcoal-based fire frequency and peak magnitude records (indicative of local fires), several differences become apparent between the lowland and mid mountain localities (Fig. 3). First, warming at ~ 16,200 cal BP and even at 14 700 cal BP did not lead to massive afforestation in the lowland; instead, a modest increase in forest cover was accompanied by the expansion of grass-steppes. A second striking difference is in fire history. The lowland site was characterised by increased fire activity from 17,800 cal BP until the LG onset (14,700 cal BP), when broad-leaved trees became dominant in this region. The onset of fire increase pre-dates the estimated disappearance of the megafauna, but appears at the time of the drastic population decrease of these megaherbivores.

It also seems that floristic diversity correlates well with vegetation openness and vegetation type; in case of the lowland site, the sequence tundra → cool steppe → cool coniferous forest steppe resulted in an increase in palynological diversity and evenness; at the mid mountain site, however, palynological diversity decreased when closed mixed leaved and taiga forests started to dominate from the LG onset (~ 14,700 cal BP). Taxa accumulation curves (Suppl. Figs. 9 and 10) also indicate a change in the local species pool at the LG onset. This has implications for the grazing megafauna as well: first, the least diverse floristically was the steppe tundra (E(T) < 20), while the forest steppe biomes were more diverse (E(T) ~ 30). On the basis of the radiocarbon dates from woolly mammoth and reindeer (Suppl. Figs. 7 and 8), a decline in these species is observed when locally tundra (steppe tundra) disappeared.

Figure 2 shows pollen-inferred July mean temperatures at Kokad using two different transfer functions^18^. While the WA-PLS reconstruction indicates that between 19,440 and 16,700 cal BP pollen-inferred summer mean temperatures were stable and fluctuated between 14.5 and 15.7 °C, the MAT based reconstruction yields lower values and a high amplitude fluctuation. A distinct cooling is present between 17,900 and 16,280 cal BP, with inferred summer mean temperatures between 7.5 and 12.7 °C. This interval coincides with HE-1 (17,850–16,200 cal BP), and the reconstructed temperature values follow the summer mean temperature values indicated by the vole fauna at the Jankovich Cave^21^ (Fig. 2). This suggests that the MAT based reconstruction is likely to be realistic. Following this event, July mean temperatures increased rapidly to 16–18 °C. Both the vole and pollen based summer temperature reconstruction demonstrate that the highest amplitude warming took place before the LG onset, around 16.2 ka cal BP in the CB and summer mean temperatures decreased only modestly during the Younger Dryas climatic reversal (Fig. 2), corroborating earlier proxy based reconstructions from the CB lowlands and SE Carpathian mountain sites^16,37–39^.

## Discussion

### The order of events: ecosystem changes in the Carpathian Basin and Europe during the last glacial termination

The multi-proxy data (Fig. 3) suggest that the collapse of the large herbivore fauna slightly preceded or coincided with the terrestrial vegetation change in the CB. In addition to the radiocarbon dated bones of woolly mammoth (Suppl. Fig. 8), fungal spores (*Sporomiella*, *Sordaria*-type (HdV55a), 1.4–230 spores cm^−2^ yr^−1^) living in the dung of large grazing mammals confirm a population decrease from 17.2 ka cal BP that likely culminated around 16.2 ka cal BP, contemporaneous with the last dated mammoth, even though the final disappearance is modelled to 13.8–15.2 ka cal BP. As demonstrated by the Kokad micro- and macrocharcoal records, regional and local fires appeared from 17.8 ka cal BP in the eastern lowland of the CB up until 14.3 ka cal BP, when the spread of temperate woody species started. A correlation is also apparent between the last ^14^C date for a mammoth and the transformation of the herbaceous vegetation at the Kokad locality. The picture is therefore less definite here than in North America, where fungal spore, charcoal and pollen studies have demonstrated that the population decrease of megaherbivores preceded the vegetation change^40^. In the CB grazing indicator fungal spores are less frequent in the LP and LG deposits, rendering this less conclusive; in addition, radiocarbon dates suggest coincidence of the change in vegetation and megaherbivore latest radiocarbon dates in case of the woolly mammoth. Overall, the data presented here suggest that a previously large herbivore population probably decreased earlier than the biome shifts took place, and thus the limiting factor for large grazing mammals was not the decreasing availability of food resources, but their intolerance of increased warmth. Furthermore, regional wildfire histories were divergent in the mountain and lowland localities, depending largely on tree cover (Fig. 3). From regional summaries it is known that wildfires were generally more frequent during the Early Holocene in EC Europe, and in conifer forest dominated regions during the LG^9,41^. This increase in fires may be explained by orbital forcing (warmer than present summers, cold winters, seasonal drought stress); however, the same studies also concluded that once temperate broadleaved forest had become established, biomass burning was high at ~ 45% tree cover and decreased to a minimum at between 60 and 70% tree cover. These data corroborate the determination of biomass burning by tree compositional change and tree cover. It was the highest at medium needle-leaved tree cover in the region.

The microfaunal changes and the vegetation changes inferred from the vole community from Jankovich and Rejtek I are the first rigorously dated cave sequences that yield information about ecosystem changes during the last glacial termination in Northern Hungary. Their merit is that they demonstrate that the point at which faunal turnover took place was not the LG onset, but the post HE-1 warming occurring at ~ 16.2 ka cal BP (see Jankovich Cave record on Fig. 2 and Suppl. Figs. 5 and 6).

Such high-resolution microfaunal records are rare in Europe. In Hungary, two cave faunal assemblages were revisited recently^4,42^, including Rejtek I and Petényi Cave in the Bükk Mts, where mollusc shells were used for dating the earlier sequences, which also contained undiagnostic Late Upper Palaeolithic artefacts, such as retouched bladelets. The dates based on mollusc shells are close to the oldest estimates given here (Suppl. Table 3), in the LG interstadial layers (11–12) of Rejtek I, and the mollusc ^14^C ages are also older, while in the Early Holocene layers there is a good agreement between the bone collagen and mollusc carbonate based ^14^C dates.

Overall, this discrepancy, plus the deposition hiatus that demonstrably took place during the LG interstadial (between ~ 14,530 and 9270 cal BP) in the Petényi Cave, and the older dates for some large grazer or predator bones in Rejtek I and Jankovich serve to warn researchers that the interpretation of various cave sediment fauna has to be treated with caution, and the best dating results and faunal based inferences can be obtained from micromammal assemblages. Furthermore, the bone and charcoal assemblages in the bottom layer of Petényi Cave (dated between 15,180 and 14,530 cal BP) indicate the development of a transitional flora and fauna (boreo-nemoral forest dominated by spruce) in the Bükk Plateau^4,23,43^ supporting the pollen records from the CB that afforestation and warming started directly after HE-1.

In Europe systematically analysed and radiocarbon dated Late Pleistocene—Early Holocene cave sediment sequences with rich bone assemblages are rare. The few that cover a similar time period are in Western France^44,45^, Spain^46^ and Ukraine^47^. In addition, a recent summary work^48^ compares ecosystem changes at a regional scale over the last 50,000 years.

The faunal turnover at 16.2 ka cal BP identified in Jankovich Cave has also been detected in two French cave sequences (Peyrazet and Coulet des Roches). In both cases, changes in the small mammal communities between the Pleistocene and Holocene were the result of a succession of climatic events starting at the end of HE-1. Several rodents that occur in temperate and forested habitats today (e.g. the garden dormouse (*Eliomys quercinus*), wood mouse (*A. sylvaticus*), bank vole (*C. glareolus*), Mediterranean pine vole (*Microtus* (*Terricola*) *duodecimcostatus*)) appeared in the middle or at the end of the LG, while species adapted to cold (e.g. arctic lemming, narrow-headed vole) gradually disappeared^44,45^. The three radiocarbon dated reindeer bones from the Peyrazet Cave gave an age range 13,835–15,410 cal BP^44^, i.e. slightly more recent than the results from Jankovich Cave, falling mainly within the Bølling interstadial. Similar faunal turnover and climatic changes were observed in the El Mirón Cave sequence^46^, where an increase in forest species diversity was detected between 18 and 11 ka cal BP, while *Pliomys lenki* (an extinct Pleistocene vole) and some other species mainly disappeared at the end of the YD.

According to Puzachenko and Markova^48^ the CB belongs to the Central European South region, where after the significant decrease in species richness of the LGM, the number of species was gradually restored to the value characteristic of the late MIS-3 between ∼ 17.5 and 13 ka BP. If local last detection times from the Rejtek I and Jankovich are compared with the regional disappearance times reported, it is possible to conclude that all Late Pleistocene species disappeared earlier from the CB than from the rest of the region. Even though the number of systematically dated cave sequences is still low in the CB, and thus later local extinction times are plausible, these relatively early local last detection times may, in all likelihood, be connected to the southern geographical position of the basin, and also to the biome mosaic that characterised this region during the LPG and LG.

In comparison with the aforementioned areas, the Grot Skeliastyi Rock Shelter in south-western Crimea indicates a different Pleistocene-Holocene faunal transition^47^. Only the large herbivore species became extinct from this assemblage, while most other taxa persisted from the Pleistocene into the Holocene without losses, a phenomenon likely to have been influenced by the Crimea’s geographic position and milder climate. Its relevance to the CB lies in the vegetation and fauna of the south-eastern lowland areas that via the Iron Gate are directly connected to the Pontic Crimean territories and contain several common taxa^49^. Many of the Pontic species originated from this climatically relatively stable area, and as the differences of the faunal records demonstrate, it is likely that Pontic species migrated into the CB during the last glacial termination period^50^.

### Plaid ecosystems reverting to equilibrium ecosystem mosaics: key to steppe fauna survival

Sommer and Nadachowski^51^ have demonstrated that faunal communities during the LGM contained a combination of cold and temperate faunal elements in the Balkans (except Greece), in SW France and in the Carpathian Region. Consequently, in these regions delayed expansion of new faunal communities in response to climate change were not influenced by delayed immigration. When the order of changes in different ecosystem components is examined^12,17,50^, a similar situation applies to certain woody and herbaceous elements of the temperate forest and forest steppe flora.

According to the Plaids and Stripes Hypothesis^52^, the main cause of the Late Pleistocene megafauna extinction was the cessation of short-term climate fluctuation during the last ice age that added a dimension of temporal complexity, which is now missing from many modern ecosystems^53^. Millennial- and centennial-scale high amplitude climate fluctuations kept ecosystems out of balance, as plant and animal species struggled to keep up with repeated shifts in their environments. Advantageous eco-physiological attributes, such as greater mobility, lower cost of locomotion, greater dietary breadth and higher metabolic efficiency allowed the Late Pleistocene fauna to flourish in such plaid settings characterised by disequilibrium. Due to frequent climatic disturbance, early successional plant communities dominated by forbs and graminoids were the key elements in supporting dense populations of megafaunal herbivores^54,55^ on immature, and thus more productive, soils. In support of this hypothesis, the transformation of the plaid ecosystems towards a striped structure during the LG and Early Holocene can be easily traced on Eurasian biome simulation maps^56^.

The pollen based biome reconstruction, microfaunal change and climate model simulations presented here all suggest that the rapid transformation of the plaid landscape took place from 16.2 ka cal BP in the CB, and the major element was the expansion of temperate and boreal woodland species and the overall gradual increase in woodland cover that did not favour mammoth and reindeer. On the other hand, if current and simulated plant biomes in the CB are examined, it is possible to see that the striped boreal and cold temperate biomes break up in the lowlands of the basin, where the so called equilibrium mosaic ecosystem (with edaphic steppes and temperate forest steppe) persisted throughout the Holocene for edaphic and hydrological reasons^49^.

This deviation from the regional trend probably had an overarching consequence during the Holocene climate stability, allowing the longer subsistence of mega-herbivore mammal species in the lowlands, as demonstrated by several studies^3,57,58^. Although climate change led to the replacement of the ungulate species spectrum due to partial afforestation, early warming around 16.2 ka cal BP was detrimental to the mammoth adapted to cold-steppe, tundra-steppe environments. As a very distant and indirect parallel, the lowlands of the CB are somewhat similar to the African savannah, where dryland ecosystems are particularly susceptible to millennial-scale boom-and-bust cycles in primary productivity^59^, and therefore plaid ecosystems are pertinent.

### Local herbivore extinctions in context of European extinction history

#### Reindeer

It is known from range modelling that the global potential range of reindeer declined by 84% between 21 and 6 kyr BP^60^. This is explicable in terms of rapid climate change, particularly after HE-1. Starting from their modern July mean temperature tolerance of < 12–13 °C and a metabolic adaptation to < 15 °C^61^, their distribution dynamics in the CB suggest that reindeer were common in the CB during the LGM (from *ca*. 23 ka cal BP) and the population had declined steeply by ca. 15.2 ka cal BP (Suppl. Table 5), with a modelled last appearance at ~ 11,860–12,550 cal BP. The range dynamics of reindeer in Europe summarized recently^62^ showed only four context-dated reindeer findings from the CB, all of which were dated between 18 and 25 ka cal BP. Recently, the Zöld Cave from the central CB^8^ yielded a reindeer bone dated to 15.4–16 ka cal BP, and further reindeer remains were associated with a charcoal date 14.9–15.3 ka cal BP. In this context, the dating results of several reindeer bones from Jankovich Cave presented here with a calibrated (2σ) age range of 15,085–20,540 cal BP (Suppl. Table 5) suggest that reindeer persisted in the basin after the LGM, and their local extinction probably occurred later than assumed by Sommer et al.^62^.

The present data suggest that the reindeer population declined considerably around 15.2 ka cal BP. Comparing this timing with the climate reconstruction inferred from pollen and chironomid, together with biome records (Figs. 2 and 3)^38^, it is possible to conclude that reindeer persisted in the cool conifer forest steppe environment of the basin for about 1000 years, and their population decline predated the emergence of cool mixed (coniferous-deciduous) forests around 14.7 ka cal BP (Fig. 2; Suppl. Fig. 11). The species was abundant during the LGM and persisted during the subsequent period, as demonstrated by the relatively large number of Late Epigravettian sites in Hungary where reindeer bones are present.

In Southern Sweden, reindeer extinction took place at the transition from open pine-birch forest to pine-deciduous dominated forest transition. In the CB this coincidence of deciduous tree expansion and decline in the reindeer population cannot be demonstrated, although both elm (*Ulmus*) and hazel (*Corylus*) were already expanding regionally in the Great Hungarian Plain at 15.2 ka cal BP without a biome shift (Fig. 2). It is likely that rapidly increasing summer temperatures had a direct effect on the local reindeer population, and that this probably left the basin as a result of its metabolic adaptation to < 15 °C. As the climate reconstructions used herein demonstrate, not only were the lowlands certainly too warm for reindeer by 15.2 ka cal BP, but also the mid mountain regions (Fig. 2). Another striking feature of the plain is that during the abrupt biome shift from a tundra to cool coniferous forest biome, the lowlands and low hills of the CB remained partially steppe covered, and this characteristic of the landscape must have helped the survival of grazers if their metabolic/physiological adaptation made it possible. The timing of the reindeer’s withdrawal from the CB agrees well with the dates of the youngest/latest Epigravettian campsites^8,63^. Even though the Epigravettian population also hunted horses by this time^8^, the coincidence of these two events suggests quarry fidelity and environmental determinism.

#### Woolly mammoth

Available summaries on European proboscidean extinction times suggest that woolly mammoth (*Mammuthus primigenius*) was present in the ice-free parts of Europe during the Weichselian Glacial until 14 ka cal BP, when its populations collapsed due to warming^64^. Moreover, the endemic European mammoth population became extinct after 24 ka cal BP and was replaced by members of a Siberian mammoth genetic clade which had been colonizing Europe since 34 ka cal BP^65^.

In the Austrian Alps, an area occupied by an extensive ice-stream network during the LGM, these animals migrated several tens of kilometres into alpine valleys during the first half of MIS 3^66^, when ice-free conditions prevailed in the major valleys. Over 230 bones have been examined in Austria, and their distribution suggests that mammoth were present in river valleys and adjacent loess covered forelands of the Alps.

Considering the two periods of apparent absence of woolly mammoth in the CB (32.5–27.4 and 24.5–22 cal BP; Suppl. Fig. 8.), the onset of the latter period coincides with a massive dust accumulation period above Greenland (see Ca^2+^ on Fig. 3) followed by two short interstadials (GI-2.1 & GI-2.2). Regarding the earlier time interval, four Greenland interstadials fall within this (GI-3-4-5.1-5.2; Suppl. Fig. 8)^34^, and it is known, mainly from loess mollusc studies, that the lowlands of the CB were covered by boreal parkland forests at these milder time intervals^67^. Furthermore, variations in glacial dust deposition on centennial–millennial timescales in the CB and Greenland were synchronous^68^. Even though the number of ^14^C dated mammoth bones is still low, if the apparent low figures for the mammoth population or indeed its absence are valid, then it is likely that the warmer and more forested periods were disadvantageous to its populations in the CB. Moreover, the European mammoth population extinction after 24 ka cal BP likely also affected the CB, where on the basis of ^14^C measurements that form part of this study, the Siberian clade’s expansion is probable after 22 ka cal BP.

### Epigravettian hunters and megafauna extinction in the CB: the relationship between human population and faunal change

Even though human activity as a result of hunting and habitat modification are often cited as the principal driving force in megafauna extinction^69^, the diversity of extinction patterns observed on different continents has led to an increasing recognition of the potential synergistic role of climate change^60^. As demonstrated by Cooper et al.^70^, in many cases the extinction of genetic clades coincided with rapid warming events in the absence of a human presence in North America. These events involved the rapid replacement of one species or population by a conspecific or congeneric one across a broad area. It appears that cold conditions were not an important driver of extinctions even in the presence of anatomically modern humans in Europe.

In the CB, Early Epigravettian groups were present during the LGM (GS-3–2.1c) and Late Epigravettian in the GS-2.1a-b and early GI-1 periods^7^. According to Lengyel et al.^71^ Early Epigravettian (26–20 ka cal BP) hunter-gatherers subsisted on reindeer and the wild horse, with reindeer predominating. A marked change was detected in the dominant quarry at the Late Epigravettian sites (20–15.2 ka cal BP), when reindeer decreased, while the wild horse became dominant, and mammoth was present again. These changes suggest that the LGM reindeer population thinned out in the CB during the last glacial termination. It has also been demonstrated that a decrease in human population of the CB took place at the end of the Late Epigravettian^8^. So far only Lovas (14–13 ka cal BP)^72^, and a stray find from Mezőlak (13.7–13.46 ka cal BP)^73,74^ are known from Transdanubia that are contemporaneous with the Epi-Magdalenian Culture of Czechia^75^ and the Arched Backed Point techno complex of Poland^76^.

These findings attest to the fact that human groups with new persistence strategies appeared in the western CB about 1000 years after the mammoth, reindeer and wild horse hunters left. Lovas provided evidence for the hunting of the Eurasian elk (*A*. *alces*) and Red deer (*C*. *elaphus*) and the use of their bones for ochre mining^77,78^. From these still fragmentary data it is possible to conclude that the Late Epigravettian population that seems to have left the CB around 15 ka cal BP had strong quarry fidelity and hunting habits, with the result that it followed the megafauna elements. Supporting this argument are the recently dated Late Palaeolithic camp sites (13–11.7 ka cal BP) further north in Slovakia, and a few in Southern Poland^79^, where the fauna is too poorly preserved to determine hunted species.

## Conclusions

In this paper the hypothesis that transitions from cold to warm intervals were briefly optimal for grazing megafauna, followed by rapid extinctions has been tested^15^. The results support this hypothesis in that the dated bone assemblages of both woolly mammoth and reindeer attest to relatively large and increasing population sizes after the LGM until c. 16.2 ka cal BP. The paleoclimate and biome reconstructions suggest that a major warming at 16.2 ka cal BP was, however, detrimental to these populations, probably due to the intolerance to increased warmth in the case of reindeer. It has also been demonstrated that in the CB, vegetation regime shifts inferred from pollen data coincided with or slightly postdated the local population decline of woolly mammoth and reindeer. Furthermore, wildfires played a role in the transformation of the vegetation at the last glacial termination, when the array of biome changes was divergent. Afforestation by cold deciduous and cool coniferous trees was rapid in the mid mountains from 16.2 kyr cal BP, while steppe tundra biomes transformed into boreo-nemoral forest steppe in the lowland localities.

When the increasing number of well dated Late Epigravettian sites and their associated fauna in the CB are compared with the biome and climate reconstruction on the basis of pollen, it is possible to infer that the reason for the disappearance of the Epigravettian hunters was the diminishing population size of their dominant quarries, while vegetation change was more gradual. Placing this in a European context, the CB behaved similarly to some south and south-west European areas, and underwent earlier (post HE-1) and more drastic faunal and human population changes in response to early warming after the LGM than more north-westerly and northerly locations in Europe.

It was found that cave faunas in general can be used to estimate local extinction times. Direct AMS ^14^C measurements on the investigated species are required; large mammal bones from the same sediment layer often give deviating (older) ages to the associated micro-mammal fauna. Since Late Pleistocene cave faunas have extensively been studied and preserved in museum collections in the CB, there is still ample opportunity to resolve the many open questions of rapid faunal changes at the last glacial termination in this region of Europe.

## Methods

### Bone sampling from the museum collections

The primary consideration in selecting remains was to choose large or medium sized mammal species or larger sized micromammals that were members of the vertebrate fauna in the Carpathian Basin during the Late Pleistocene but which became extinct in the latest Pleistocene or in the Holocene. Since the aim was to determine the probable age of disappearance, preference was given to bones of the same species from all layers in the sequence, where possible.

### Radiocarbon dating, age-depth modelling

The selected bones were first subjected to physical preparation, including surface removal using a Dremel device, followed by grinding and sieving. Then 600 mg of the 0.5–1 mm fraction was placed into a test tube to perform acid–base-acid treatment (ABA), using 0.5 M HCl and 0.1 M NaOH reagent, rinsing the sample with ultrapure water in between. The pH of the samples was then adjusted to 3 and the test tubes were put in a block heater at 75 °C for 24 h to gelatinize the collagen. The liquid gelatine samples were then filtered using a 2 μm glass fibre filter and freeze dried for two days. For combustion, a ~ 4 mg gelatine sample together with MnO_2_ reagent was placed to a glass combustion tube, which was, after sealing, heated to 550 °C^80,81^. The CO_2_ gas thus obtained was then purified and reduced to solid graphite, applying a customized sealed tube graphitization method^82^. The ^14^C measurements were performed using the EnvironMICADAS AMS instrument at the ICER laboratory^83^.

^14^C ages were calibrated into calendar years using Calib Rev. v. 8.2html software and the Intcal20 calibration curve^35^. Age-depth modelling for the cave sediment sequences was performed with the Bacon package in R using Bayesian probability statistics^84^. The woolly mammoth and reindeer radiocarbon dates presented in Supplementary Tables 4 and 5 were calibrated against the IntCal20 dataset using OxCal version 4.3 and incorporated within a single Phase model in OxCal version 4.3 in order to provide an estimate of the last appearance dates^32,85^. The ‘Phase’ command is a grouping model. It assumes no geographic relationship between samples, and that the ages represent a uniform distribution between a start and end boundary. The posterior distributions allowed the determination of probability distribution functions (PDFs) for the beginning and end of the phase. Modelled ages are reported here at the 95% probability range in thousands of calendar years BP (years; relative to AD 1950). An alternative model was also used to estimate extinction times of woolly mammoth and reindeer. The GRIWM model^33^ inversely weights the contribution of each dated record to determine the terminal date. Equations and further details of the method are described in the Supplementary Material.

### Re-analysis and re-plotting of the mammal bone assemblages

Bone assemblages were re-analysed for taxonomic inconsistencies by Piroska Pazonyi. Stratigraphic diagrams of the vole and other small mammal faunas were plotted in Psimpoll 2.27 and edited in Corel Draw 16.

### Quantitative climate reconstruction (pollen & vole thermometers)

The method of the pollen-based summer mean temperature (T_JJA_) reconstruction from Kokad Mire was described in detail in Magyari et al.^18^ In brief, the European Modern Pollen Database (EMPD) was used as training set^86^. 209 terrestrial pollen types were used for the summer mean temperature reconstruction, and this was further reduced to the dominant 43 taxa for the modern analogue technique reconstructions. The training set has 2687 modern pollen samples, of which 1240 are located below 600 m above sea level. Modern T_JJA_ (mean air temperature for June, July and August) for each site was included in the EMPD climate data file. Quantitative pollen-based transfer functions were developed for T_JJA_ using weighted averaging-partial least squares (WA-PLS) regression^87^ with five components. Leave-one-out cross-validation^88^ was employed to evaluate the model performance and estimated performance statistics such as coefficient of determination (r^2^) between measured and predicted values, root mean square error of prediction (RMSEP) and maximum bias for each WA-PLS transfer function. The surface pollen data was transfomed to square-roots to reduce the noise of the data^89^, a randomization t-test ^90^ was applied to select the most appropriate WA-PLS component for TJJA reconstructions, and sample-specific standard errors were calculated for the reconstructions, using a bootstrapping procedure with 1000 cycles^91^. The WA-PLS transfer functions and associated T_JJA_ reconstructions were performed using the RIOJA package in R^92^. The statistical significance of the WA-PLS based T_JJA_ reconstructions was assessed using an approach involving 999 randomizations developed by Telford and Birks^93^, and these significance tests were conducted using the R package PALAEOSIG^93^. An alternative transfer function, the modern analogue technique (MAT) was also used to derive T_JJA_. This method does not require real calibration and is based on a comparison of past pollen assemblages to modern assemblages. The similarity between each fossil and modern pollen assemblage is evaluated using the chord distance metric^94,95^. In this study, the six modern pollen spectra that had the smallest distance were considered the best modern analogues of the given pollen spectrum, and were subsequently used for the reconstruction. If the chord distance was above a threshold defined by the Monte-Carlo method^94^, the modern sample was considered a bad analogue, and was not taken into account in the reconstruction. Estimates of climatic parameters were obtained by taking a weighted average of the values for all selected best modern analogues, where the weights used were the inverse of the chord distance. MAT based T_JJA_ reconstruction and statistical tests were run in R using the RIOJA package. In this study the focus was on the reconstruction of a single climatic parameter, T_JJA_. The aim of the reconstruction is to compare the pollen-based T_JJA_ record with the extinction times of both mega and microfauna elements and the pollen based TJJA reconstruction were also compared with the vole-thermometer based T_July_ reconstruction from the Jankovich Cave and Rejtek Rock Shelter^21,23^. The method is a paleozoogeographical calculation based on the principle of actualism; it is used to determine the July (summer) temperature of the accumulation period of different samples^21,96^. The present-day optimum temperature for the distribution of certain vole-species is given in Jánossy and Kordos^23^. These are 15 °C for *Clethrionomys*, 17.5 °C for Arvicola, 21 °C for *Microtus arvalis*, 19 °C for *Microtus agrestis*, 10 °C for *Lasiopodomys* (*S.*) *gregalis*, 12.5 °C for *Microtus oeconomus* and 7.5 °C for *Dicrostonyx*. After the multiplication of the values of mean July temperature by the percentage of the species in question compared to other vole-species, these values were summed up and divided by 100. *Microtus nivalis* was not taken into account because it is a mountain species, so its distribution is not zonal^21,23^.

### Biome reconstruction

For the pollen assemblage based biome reconstructions the technique of Prentice et al.^97^ was used, as improved further by Tarasov et al.^98^. The first step was the assignment of the pollen taxa to plant functional types (PFT). The biome-PFT-taxon matrix published in Allen et al.^99^ was used here. Plant functional types occupy specific bioclimatic spaces that can be assigned to one or several biomes. The biome-PFT matrix is a list of biomes, indicating which PFTs are characteristic of each biome. The next step is the calculation of biome affinity scores^97^. This equation sums up the square roots of pollen percentages within a PFT and sums the affinity scores within a biome characterised by a set of PFTs. Eventually, the pollen sample is assigned to the biome with which it has the maximum affinity. The threshold value above which a taxon is considered is 0.5%. If the arboreal pollen sum is < 70%, the temperate forest biome is replaced by wooded steppe^99^. In this paper, the stratigraphic plot of the main biome affinity scores and the assigned biomes is presented.

## Supplementary Information

Supplementary Information.

## Data Availability

The data that support the findings of this study are openly available in Mendeley Data at http://dx.doi.org/10.17632/9kwbj5y54j.1.
