# Supplementary material for: Mammal extinction facilitated biome shift and human population change during the last glacial termination in East-Central Europe
Source: Sci Rep. 2022 Apr 26;12:6796. doi: 10.1038/s41598-022-10714-x (PMC9043214; doi:10.1038/s41598-022-10714-x)
Supplement: Supplementary file 1 — Supplementary Information. [file 41598_2022_10714_MOESM1_ESM.pdf]

Study sites

Jankovich Cave (Bajót, Gerecse Mts., North Hungary)

Jankovich Cave is situated near Bajót (North Hungary), 330 m a.s.l. within the Upper Triassic Dachsteinian Limestone of the eastern slope of Öreg-kő Hill. It consists of a 27 m long north-south oriented outer section, a 15 m long northwest-southeast oriented inner corridor, and a dome (Fig. 1.). An almost 8 m wide chimney opens up to the top of the hill near the entrance.

Several trenches (also called “blocks”) were excavated within the cave[1]. According to Hillebrand[2], the stratigraphic section at the entrance consists of: a sterile yellow clay at the bottom, a yellow clastic clay with plentiful reindeer (*Rangifer tarandus*) bones and microfauna, a sterile yellowish-grey clay, and a brown humus on the top with Bronze Age ceramics. A red clastic clay layer was also found in the inner part of the cave intercalated between the sterile yellow clay at the bottom and the bone-rich yellow clastic clay. It lacks microfauna, but contains plentiful cave bear (*Ursus spelaeus*) bones fragments.

The main portion of the material we studied (212 specimens) was excavated by J. Hillebrand between 1913 and 1925, and later studied by Lambrecht and Kordos[3]. The bone-rich sediment was probably located in the middle and inner parts of the cave. An excavation carried out by D. Jánossy and S. Bököny in 1956 opened six “blocks” (ie. trenches), of which block II yielded a considerable faunal assemblage. A total of 47 specimens of trench 2 of 1956 also were involved in this article. All specimens from the site are stored at the Palaeontological Collections of the Mining and Geological Survey of Hungary.

Pollen analysis of the sediments was also done by Mihálz-Faragó[1]. The quantity of the recovered pollen was poor, and contained only a few *Pinus*, *Tilia*, *Betula*, *Salix*, *Alnus* and Poaceae grains unsuitable for quantitative analysis.

Hollendonner studied the charcoals and reported the presence of elm (*Ulmus*), ash (*Fraxinus*) and oak in the "lower" layer group, and oak (*Quercus*) and pine (*Pinus*) in the "middle” layer group. The age and exact location of the charcoals in the sediment is unknown.

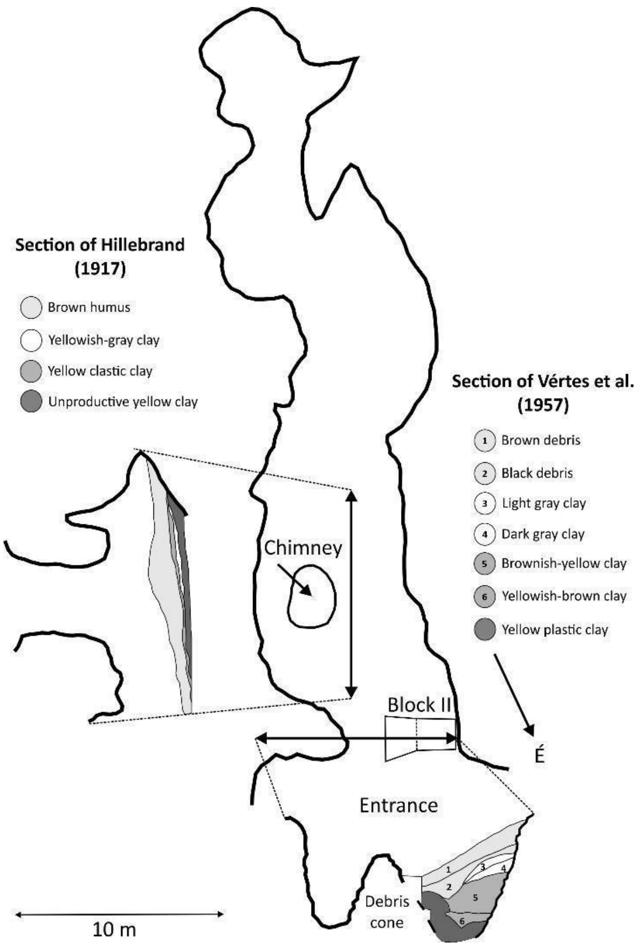

Figure 1. Layout (A) and schematic sections (B and C) of the Jankovich Cave (modified after Hillebrand[2] and Vértes et al.[4]

Rejtek I. Rock Shelter

Rejtek I. Rock Shelter is situated in the central Bükk Mountains, east of the village Répáshuta in a valley. The rock shelter opens at the eastern slope of the valley [5]. At the north-western end of the Szarvaskő Hill a 10-15 metres high rock mass can be found under which there are several rock shelters and clefts. One of them is the Rejtek I. Rock Shelter (Figure 2). It is formed in the well-stratified Middle Triassic Répáshuta Limestone. Its entrance facing west lies 8-10 metres above the valley-bottom. Its width is 9 metres in N—S direction, its ceiling height 1.60 m, and the cavity penetrates 2.50 metres into the parent rock. Behind the rock shelter there is a semi-circular lateral branch that has two entrances (Fig.2). Jánossy and Kordos [5] gives a detailed description of the excavation trenches (I-II-III) and also describes the harmonization and renumbering of sediment layers obtained from different sediment trenches. The site was first excavated in 1957 followed by a second field campaign in 1958 [6]. An exceptional value of the site is the availability of a detailed investigation on the charcoal remains by Stieber[7] and its mollusc fauna analyses by Krolopp [5] and Sümegi et al.[8]. Earlier studies furthermore emphasized that the sequence of layers in this rock shelter offers a great possibility to study the Pleistocene-Holocene transition of the vole fauna. The re-analysis of the mollusc fauna, including radiocarbon dating, and the charcoal material [8] suggested that sediment accumulation was nearly continuous from 13,000 ^{14}C BP, with a break in the Younger Dryas until the Sub-Boreal. Our results suggest that the bone assemblages are mixed in the Holocene layers, therefore we did not interpret faunistic changes in these sediment layers. During the Younger Dryas we did not find hiatus in the sediment, our age-depth model is continuous and several bones were dated to this period. The study of the archaeological finds identified a few undiagnostic artefacts from the Pleistocene layers and a Mesolithic trapeze from the Early Holocene [9]. July mean temperatures were also estimated by the vole thermometer method and using our new age-depth model derived cal BP year intervals, the results are:

200-220 cm = 13085-14157 cal BP = 17.2 °C

180-200 cm = 12347-13085 cal BP = 18.3 °C

160-180 cm = 11549-122347 cal BP = 17.3 °C

140-160 cm = 10851-11549 cal BP = 18.3 °C

There is no reconstruction above 140 cm in Block III [5]. Overall the vole thermometer suggests about 1 °C fluctuation in July mean temperatures over the late glacial period, and generally 2-3 °C lower warmest month mean temperatures than today (between 17.2-18.3 °C)

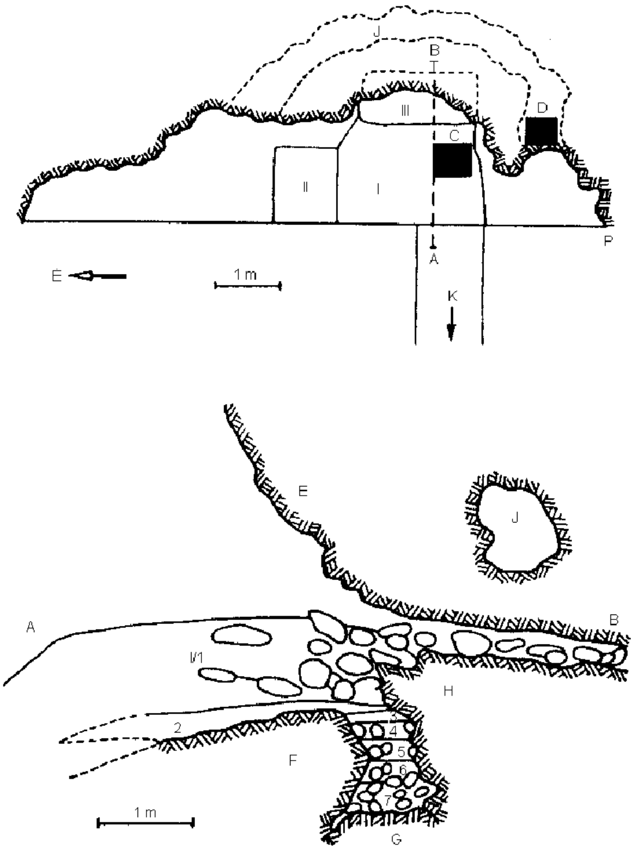

Figure 2. Sketch map of the Rejtek I. Rock Shelter and longitudinal section of the excavation (A-B) with the excavation blocks [10]. I-III: excavation blocks; 1-7: vertical layers; C&D: test holes in 1957-1958; E: rock wall; F& G: rock bottom and blocks of stones; H: rear rock wall and stone block; J: lateral branch; P: starting point of measurement.

Pollen sequences

In this study we used earlier published pollen records covering the Last Glacial Termination (20-9 ka cal BP) in the Carpathian Basin. ***Taŭl dintre Brazi*** (***Lake Brazi***) is an alpine lake that was formed in a glacial basin around 15,700 years ago in the Retezat Mts (South Carpathians, Romania)[11,12]. The small lake (~0.5 ha; 1740 m a.s.l.; max. 1.1 m water depth) is surrounded by Norway spruce (*Picea abies*) and Arolla pine (*Pinus cembra*) forest today with Dwarf pine (*P. mugo*) thickets growing on the eastern lakeshore and floating moss carpet. Its significance lies in the extreme high resolution pollen analysis and dating of this locality. Its age-depth model is based on 22 ^{14}C AMS dates and the Late Glacial part of the pollen record was studied in each cm for pollen resulting in a time resolution 20-100 yr per sample depending on the sediment accumulation rate. Apart from pollen, plant macrofossils, diatoms, Cladoceran shells and chironomids were also studied in its deposit together with a detailed geochemical study [13–18]. A chironomid-based temperature reconstruction is also available from this record [14]. ***Lake St Anne*** (950 m a.s.l.) is set in the continental Eastern Carpathians in a nemoreal beech forest. It's a cold microclimate volcanic crater lake today, formed by one of the latest eruption of Ciomadul volcano about 27,200 cal BP [19]. The size of the lake is ~189,900 m2; maximum water depth is ~6 m, mean depth is ~3.1 m, mean width is ~310 m. Its pollen sequence is continuous since 26,500 cal BP with the last glacial termination part published by Magyari et al.[20,21]. The area supported regionally diverse warm micro and meso-habitats during the Last Glacial maximum. ***Nagymohos Peat Bog*** (300 m a.s.l.; 3.2 ha) is situated in the foothill zone of the Northern Carpathians. Today nemoreal oak (*Quercetum petraeae-cerris*) and oak-hornbeam (*Querco petraea-Carpinetum*) forests cover the slopes around the peat bog that is dominated by *Eriophoro vaginato-Sphagnetum* vegetation and downy birch (*Betula pubescens*). The pollen record extends back to c. 27,000 cal BP and about 1.1 m thick peat accumulated in this basin between 14.7-26.95 ka cal BP [21–23]. The plant macrofossil record from this site demonstrates that during the LGM and last glacial termination forest tundra vegetation was present locally with arolla pine (*P. cembra*) tree stands growing on the mire surface in the lower part of the profile. ***Kokad mire*** (112m a.s.l.) is located in the Érmellék geographical region of the eastern Great Hungarian Plain. Rich in abandoned paleochannels, this region has been a mire land until the 1960's when the area was canalised. During the Late Quaternary this region was fed by rivers that drained the Northeast Carpathians and the Apuseni Mountains. Before drainage, sand dune valleys were occupied by shallow eutrophic ponds, sedge and reed dominated mires, swamps and wet meadows in this region. Dune tops originally supported steppe oak forests (*Melampyro debreceniensi-Quercetum roboris* and *Festuco rupicolae-Quercetum roboris*) or sand steppes (*Festuco vaginatae-Corynephoretum*) depending on the ground water level. The 5.5 m long sediment in this mire extends back to c. 20 ka cal BP. Pollen accumulation is continuous in this sequence throughout the Late Pleniglacial and Holocene. Apart from pollen, plant macrofossils were also studied from this profile [18,24] and a pollen based July mean temperature reconstruction was also presented that is also used in this study. The most prominent feature of the record was the early post last glacial maximum (LGM) establishment (17,700 cal BP) and expansion (14,700 cal BP) of elm (*Ulmus*) and hazel (*Corylus*) supporting the phylogeographical evidence for extra-Mediterranean refugia in the Pannonian Basin. Magyari *et al.* (2019) studied several loess and lake archives together with the Kokad pollen sequence and confirmed that in this region of Europe the warming after Heinrich event 1 (around 16,200 cal BP) had similar amplitude to the late glacial warming.

Macrocharcoal analysis – methods and Lake St Anne core SZA-2013 results

The method of the macrocharcoal analyses in case of Kokad Mire are described in Vincze et al. (2019)[18]. In case of Lake St Anne macrocharcoal analyses were performed by Ildikó Vincze on core SZA-2013, but these data have not yet been published. Therefore, we describe the method we used for data analysis. Core SZA-2013 has an independent radiocarbon chronology[25] and pollen analysis was carried out on a separate core, SZA-2010, which was published earlier[20]. Sample preparation and counting followed standard techniques[18]. Macrocharcoal concentrations (particles cm^{-3}) were transformed into charcoal influx (CHAR, particles cm^{-2} yr^{-1}) based on the age-depth model using the CharAnalysis software[26]. Considering the long temporal window and to ensure the datasets of each site were treated in similar way and results are comparable, we used a constant resampling interval of 40 years per sample for calculating charcoal influx (or accumulation rate, $CHAR_i$). Thereafter, CHAR was decomposed into background ($C_{background}$) which should reflect regional biomass burning and secondary charcoal deposition and peak ($CHAR_{peak}$) components, which reflects local fire events. Background charcoal ($C_{background}$) was modelled using a Lowess smoother robust to outliers with a 1000-year time smoothing window. The peak component ($CHAR_{peak}$) was calculated by subtracting the $C_{background}$ from $CHAR_i$. Potential fire episodes ($CHAR_{fire}$) are represented by values of $CHAR_{peak}$ exceeding the 90th percentile thresholds of the modelled local-noise distribution, which are then screened with a minimum-count test[26]. The distributions of fire return intervals (years per fire - FRIs) is described by the mean and median FRI and is used here to characterize changes in fire regimes over time. Note that charcoal fragments were absent in the sample between 13 800 and 24 300 cal BP. Results are presented in the figure below.

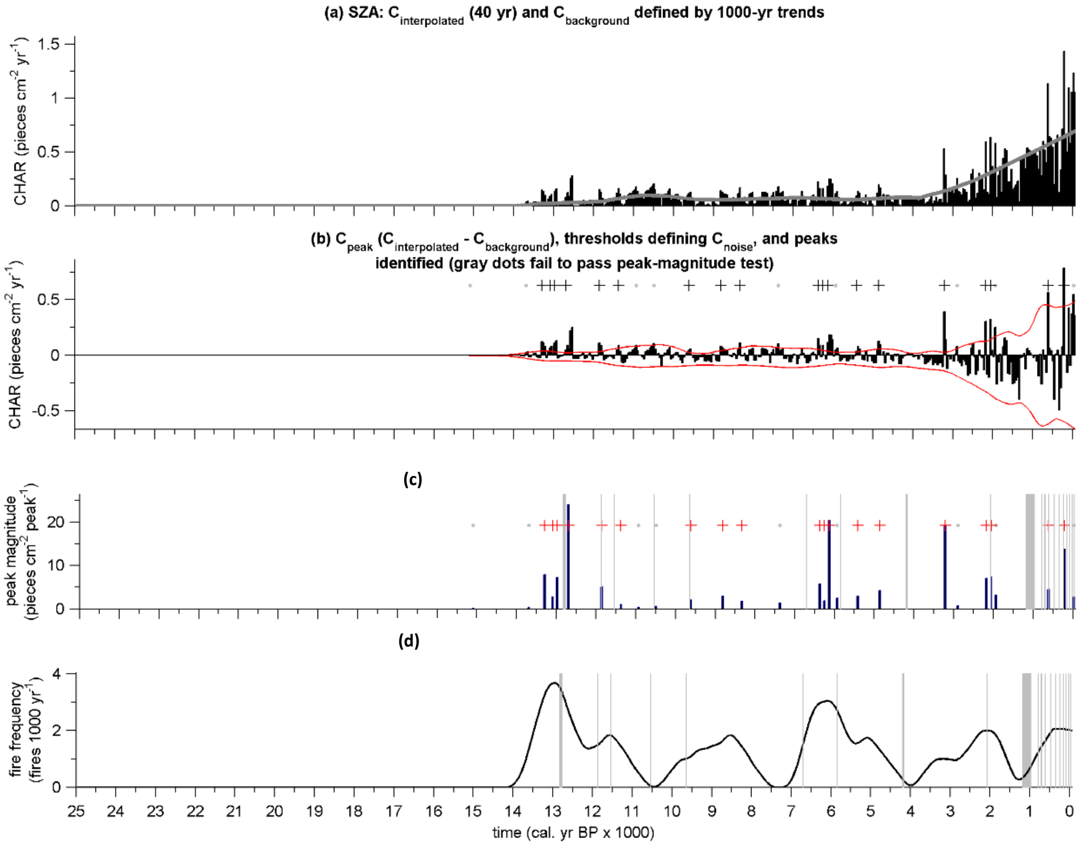

(a) Macrocharcoal accumulation rate (CHAR, pieces cm^{-2} yr^{-1}) of Lake St Anne (SZA-2013) record interpolated for 40-yrs ($C_{interpolated}$) and $C_{background}$ defined by 1000-year window, ***(b)*** C_{peaks} and thresholds defined by C_{noise} and peaks (grey dots) which failed to pass peak-magnitude test, ***(c)*** peak magnitude (pieces cm^{-2} $peak^{-1}$) and ***(d)*** fire

frequency (fires 1000 yr^{-1}). Grey lines in the bottom figures indicate the interpolated intervals for missing samples.

Estimation of extinction times for reindeer (*Rangifer tarandus*) and woolly mammoth (*Mammuthus primigenius*) and reindeer
https://doi.org/10.21203/rs.3.rs-778658/v1

1. OXCAL phase model estimates

The woolly mammoth and reindeer radiocarbon dates presented in Supplementary Tables 4 and 5 were calibrated against the IntCal20 dataset using OxCal version 4.3 and incorporated within a single Phase model in OxCal version 4.3 in order to provide an estimate of the last appearance dates[27,28]. The 'Phase' command is a grouping model. It assumes no geographic relationship between samples, and that the ages represent a uniform distribution between a start and end boundary. The posterior distributions allowed us to determine probability distribution functions (PDFs) for the beginning and end of the phase. Modelled ages are reported here at the 95% probability range in thousands of calendar years BP (years; relative to AD 1950; Fig. 1). Based on this method the phase end is estimated between **16 150 - 12 690** cal BP (with a median age of **15 210**) **for mammoths**, and **14 480 - 8 300** cal BP (with a median age of **12 550**) **for reindeer** (Fig. 1). However, these results should be treated with caution since our data is sometimes clustered or spaced widely within the observation period.

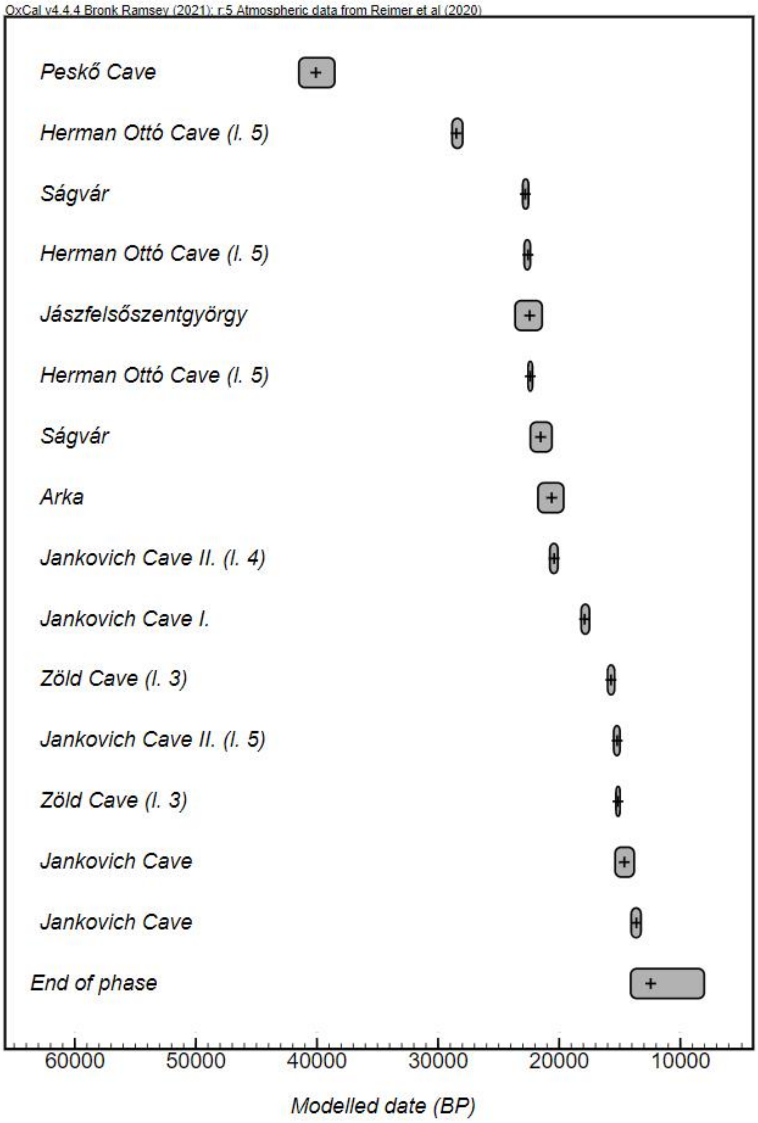

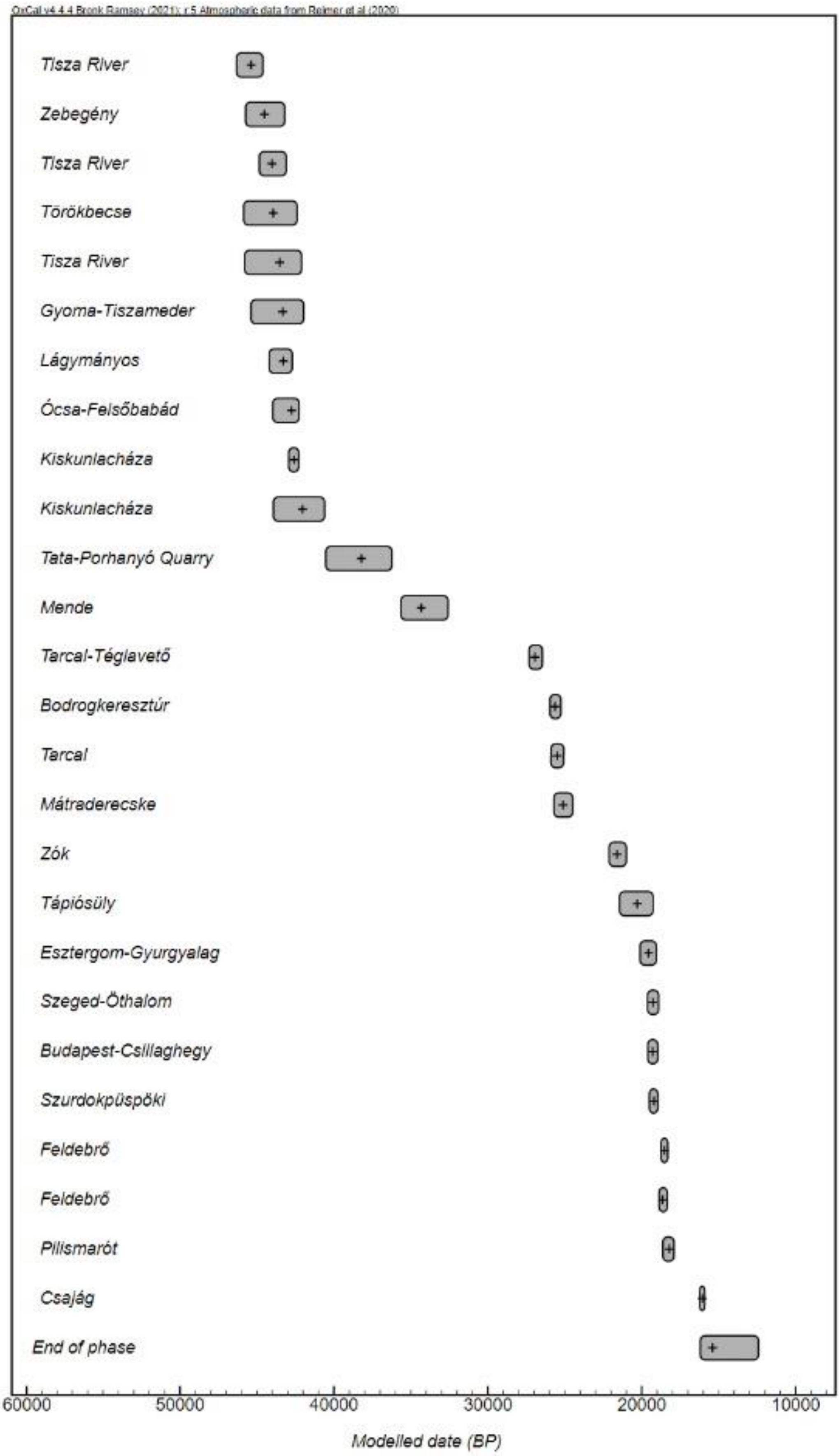

Figure 1 *OXCAL Phase estimates of reindeer (Rangifer tarandus) and mammoth (Mammuthus primigenius) extinction times in the Carpathian Basin, East-Central Europe*

2. The GRIWM method

According to McInerny et al. (2006)[29], given a set of radiocarbon dates or a series of sightings of a taxon, the probability (*p*) of finding another record can be estimated from the previous sighting rate n/t_n, where *n* is the number of records and t_n is the initial period of observations (which can be defined as the difference between the oldest and youngest records). The equation for the calculation is the following:

eq(1) $$p = \left(1 - \left(\frac{n}{t_n}\right)\right)^{t_d},$$

where t_d is the time since the last observation. For this and the following computations, we developed several easily applicable functions within the R programming environment. A specific probability value can be determined by the prob(t_d, t_n, n) function with the supplementary script[1].

A terminal date for a given taxon can be inferred by setting a threshold probability to a chosen value (e.g., $\alpha = 0.05$) and iterating until $p \leq \alpha$ and subtracting the resulting time since observation from the youngest record. Such a date can be calculated using the term(t_n, n) function within the supplementary R script[1]. Using the radiocarbon data presented in the current paper, we calculated a terminal age of 12 757 years for mammoths (that is 3 251 years after their last known record at 16 008 cal BP), and 9 219 years for reindeer (that is 4 366 years after their last known record at 13 585 cal BP; see Fig 2). These data were based on 29 observations from a period of 31 493 years in the case of mammoths, and 18 observations from a period of 26 243 years in the case of reindeer (Fig. 2).

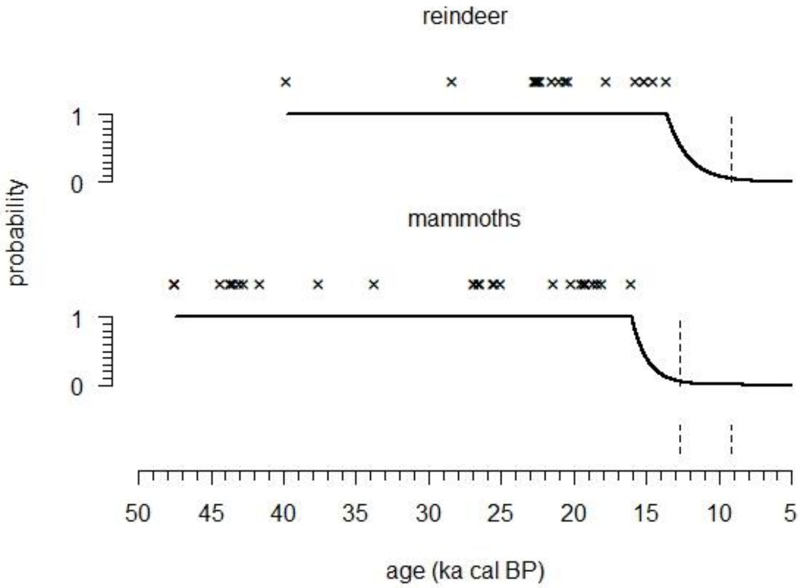

Figure 2 *Probability values of detection and the estimation of possible extinction times using the method of McInerny et al. (2006) for reindeer (Rangifer tarandus) and mammoth (Mammuthus primigenius) in the Carpathian Basin, East-Central Europe*

Bradshaw et al. (2012)[30] modified the method of McInerny et al. (2006) by inversely weighting the contribution of each dated record to the terminal date (ϑ) depending on its temporal distance from the most recent record (t_1), assuming that younger records would be more influential on the sighting rate as extinction is approached. They calculated a terminal date for the two most recent data ($k = 2$), and then iteratively increased the number of used observations during each of the following iterative steps by one, until they reached the total number of observations ($k = n$) with a series that included the oldest record as well. For every series of the youngest *k* observations, they calculated a weight as well using the following equation: $\omega_k = 1 / (t_k - t_1)$, where t_k is the oldest observation in a particular series. Then they determined the final weighted terminal age ($\vartheta\omega$) using the following equation:

eq(2) $$\theta_\omega = \sum_{k=2}^{n} \omega_k \theta_k \;/\; \sum_{k=2}^{n} \omega_k$$

The inverse weighted McInerny et al. (2006) terminal ages can be calculated using the IWM(sightings) *function* within the supplementary R script[1], where sightings is a numeric array of radiocarbon ages. Using the radiocarbon data

presented in the current paper, we estimated an extinction date of **13 830 cal BP for mammoths** (that is 2 178 years younger than their last record), and **11 860 cal BP for reindeer** (that is 1 725 years younger than their last record; see Fig. 3).

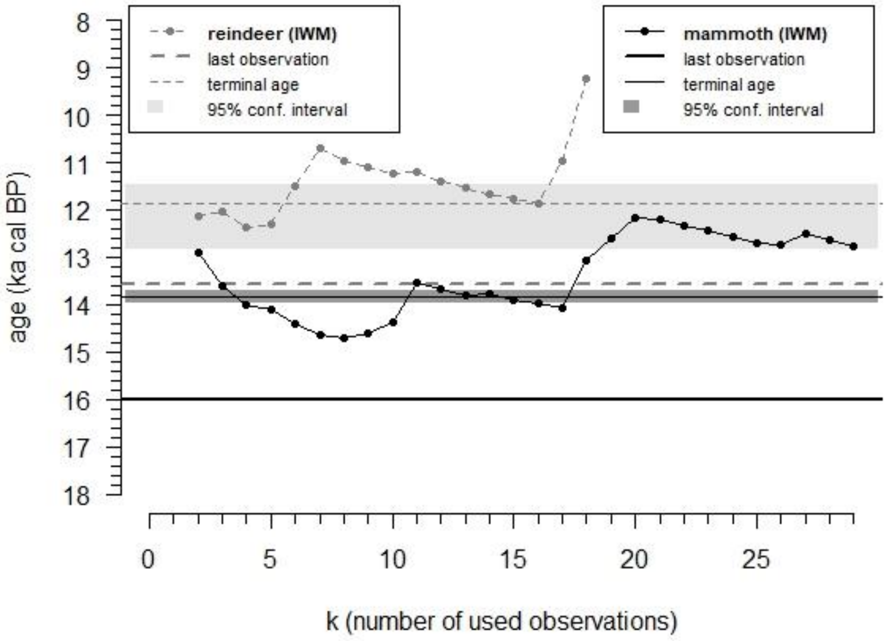

***Figure 3** Extinction time estimations for mammoths and reindeer using the Gaussian resampled, inverse weighted McInerny et al. (2006) method developed by Bradshaw et al. (2012) called the GRIWM method; inverse weighted terminal ages and best estimates of possible extinction times are shown with confidence intervals for the Carpathian Basin (East-Central Europe)*

One problem with these estimations remains that they do not consider uncertainty in radiometric dating. To address this issue, Bradshaw et al. (2012) resampled each radiometric date 10 000 times from a Gaussian distribution (because radiometric errors tend to follow a normal distribution), then calculated a terminal age for each iteration. By excluding the lower and upper 2.5% of those results, we can define a 95% confidence interval for the above calculated extinction date. This Gaussian-resampled, inverse-weighted McInerny et al. method can be determined using the GRIWM(sightings, sdevs) function within the supplementary R script[1]. In the case of our data, a confidence interval between **13 717 - 13 960 cal BP** can be determined for the extinction of mammoths and another between **12 814 - 11 471** cal BP for the reindeer (Fig. 3).

[1]Virág, A., Szabó, B., (2022): Functions for calculating terminal ages within the R programming environment from a series of radiocarbon dates by the GRIWM method. https://github.com/paleoscript/GRIWM

Supplementary Table 1 AMS ^{14}C dates obtained on fossil bones from Rejtek I Rock Shelter and Jankovich Cave and used for age-depth modelling of the sediment accumulation at both sites. Bone samples were taken from the collection of the Hungarian Natural History Museum in case of Rejtek I Rock Shelter; the site was excavated by Dénes Jánossy[6], its fauna was first published by Jánossy and Kordos[5], later revised by Jánossy[10]. Jankovich Cave was excavated several times [2–4]. The material studied in this paper comes from the 1950 excavation led by László Vértes (Block II)[1] and the 1956 excavation led by Dénes Jánossy and László Vértes. All specimens from the site are stored in the Palaeontological Collections of the Mining and Geological Survey of Hungary.

Species	Locality (layer)	C-14 age (BP)	±	Calendar age 1σ (cal BP)	Calendar age 2σ (cal BP)
**Rejtek I. Rock Shelter**					
Sus scrofa	Rejtek, Block II, 90-120 cm (8)	8768	50	9558-9927	9685-9890
Ochotona	Rejtek, Block III, 140-160 cm (9)	10243	65	11714-12238	11915-12090
Meles meles	Rejtek, Block III, 140-160 cm (9)	9727	50	11077-11242	11129-11219
Arvicola terrestris	Rejtek, Block III, 140-160 cm (9)	9594	53	10746-11155	10788-10973
Arvicola terrestris	Rejtek, Block III, 160-180 cm (10)	10392	62	12028-12434	12146-12397
Cricetus cricetus	Rejtek, Block III, 160-180 cm (10)	10125	60	11591-12016	11616-11840
Microtus arvalis	Rejtek, Block III, 180-200 cm (11)	11527	71	13206-13489	13302-13433
Cricetus cricetus	Rejtek, Block III, 180-200 cm (11)	10342	61	11952-12420	12063-12240
Microtus arvalis	Rejtek, Block III, 220-220 cm (12)	11792	76	13466-13757	13553-13725
Microtinae	Rejtek, Block III, 220-220 cm (12)	11676	71	13349-13643	13439-13566
Lasiopodomys (Stenocranius) gregalis	Rejtek, Block III, 220-220 cm (12)	11496	72	13181-13472	13278-13420
**Jankovich Cave**					
Ochotona sp.	Jankovich Cave, Block II, 110-130 cm (5)	12867	73	15216 - 15489	15130 - 15638
Rangifer tarandus	Jankovich Cave, Block II, 110-130 cm (5)	12753	77	15084 - 15309	14885 - 15496
Spermophilus sp.	Jankovich Cave, Block II, 130-160 cm (6)	13106	74	15590 - 15872	15401 - 15992
Dicrostonyx sp.	Jankovich Cave, Block II, 130-160 cm (6)	13765	95	16457 - 16821	16314 - 16973
Ochotona sp.	Jankovich Cave, Block II, 150-170 cm (7)	13535	79	16165 - 16430	16047 - 16588
Ochotona sp.	Jankovich Cave, Block II, 170-200 cm (8)	13677	78	16334 - 16630	16232 - 16813
Ochotona sp.	Jankovich Cave, Block II, 200-220 cm (9)	13967	85	16790 - 17106	16594 - 17243
Dicrostonyx sp.	Jankovich Cave, Block II, 220-250 cm (10)	14454	90	17483 - 17749	17363 - 17897
Ochotona sp.	Jankovich Cave, Block II, 220-250 cm (10)	14378	90	17389 - 17658	17224 - 17814

Supplementary Table 2 All AMS ^{14}C dates obtained on bones from Jankovich Cave; dated material was in all case fossil bone. Recommended values for C/N ratios of bulk bone or tooth range from 2.9 to 3. 31,32</sup> with collagen yields of 1% or greater [33].

**Species**	**Dated material**	**Locality (layer)**	**Collagen (%)**	**C/N ratio**	**C-14 age (BP)**	**±**	**Calendar age 1σ (cal BP)**	**Calendar age 2σ (cal BP)**
Cricetus cricetus	tubular bone (1)	Jankovich Cave, Block II. 0-30 cm (1)	9.3	3.3	9502	54	10679 - 10796	10588 - 10632
Ochotona sp.	mandible (1)	Jankovich Cave, Block II. 30-60 cm (2)	11.0	3.4	14295	93	17264 - 17553	17109 - 17670
Ochotona sp.	mandible (1)	Jankovich Cave, Block II. 60-90 cm (3)	7.5	NA	9679	100	10798 - 10851	10733 - 11245
Lasiopodomys (Stenocranius) gregalis	mandible (3)	Jankovich Cave, Block II. 90-110 cm (4)	6.3	3.1	14372	92	17378 - 17653	17210 - 17808
Ochotona sp.	mandible (2)	Jankovich Cave, Block II. 90-110 cm (4)	8.1	3.3	11297	65	13086 - 13208	13059 - 13280
Rangifer tarandus	tubular bone (1)	Jankovich Cave, Block II. 90-110 cm (4)	3.6	3.3	16894	123	20210 - 20537	20043 - 20683
cf. *Rupicapra rupicapra*	bone (1)	Jankovich Cave, Block II. 90-110 cm (4)	6.8	3.2	13243	76	15796 - 16041	15680 - 16167
Ochotona sp.	mandible (2)	Jankovich Cave, Block II. 110-130 cm (5)	6.6	3.3	12867	73	15216 - 15489	15130 - 15638
Rangifer tarandus	phalange (1)	Jankovich Cave, Block II. 110-130 cm (5)	3.7	3.4	12753	77	15084 - 15309	14885 - 15496
Spermophilus sp.	mandible (2)	Jankovich Cave, Block II. 130-160 cm (6)	5.1	3.4	13106	74	15590 - 15872	15401 - 15992
Dicrostonyx sp.	mandible (3)	Jankovich Cave, Block II. 130-160 cm (6)	5.5	NA	13765	95	16457 - 16821	16314 - 16973
Ochotona sp.	mandible (3)	Jankovich Cave, Block II. 150-170 cm (7)	6.8	3.3	13535	79	16165 - 16430	16047 - 16588
Equus sp.	incisor (1)	Jankovich Cave, Block II. 150-170 cm (7)	6.8	3.3	13967	89	16783 - 17110	16585 - 17258
Ochotona sp.	mandible (4)	Jankovich Cave, Block II. 170-200 cm (8)	6.8	3.3	13677	78	16334 - 16630	16232 - 16813
Ochotona sp.	mandible (3)	Jankovich Cave, Block II. 200-220 cm (9)	6.6	3.3	13967	85	16790 - 17106	16594 - 17243
Dicrostonyx sp.	mandible (4)	Jankovich Cave, Block II. 220-250 cm (10)	5.9	3.4	14454	90	17483 - 17749	17363 - 17897
Ochotona sp.	mandible (3)	Jankovich Cave, Block II. 220-250 cm (10)	7.6	3.3	14378	90	17389 - 17658	17224 - 17814
Ochotona sp.	mandible (3)	Jankovich Cave, Block II. 250-270 cm (11)	7.0	3.3	18544	141	22261 - 22565	22002 - 22741
Ochotona sp.	mandible (5)	Jankovich Cave, Block I. 0-20 cm	6.5	3.3	12987	72	15380 - 15671	15270 - 15788
Rangifer tarandus	humerus (1 fragment)	Jankovich Cave, Block I. 20-40 cm	10.4	3.2	14613	88	17672 - 17911	17555 - 18014
Ursus spelaeus	incisor (1)	Jankovich Cave, Block IV. 0-0.3 cm (1)	8.1	3.2	40000 BP<			

Supplementary Table 3 All AMS ^{14}C dates obtained on bones from Rejtek I Rock Shelter. The dated material was in all cases fossil bone. On the right side of the table AMS ^{14}C dated fossil mollusc shells are [8] shown with the deviation compared to the new radiocarbon dates in the last column. The mollusc shell dates are generally younger than the bones in the Early Holocene layers. In contrast, the mollusc dates in the lowermost layer of Block III are older than the bone dates. Recommended values for C/N ratios of bulk bone or tooth range from 2.9 to 3. [31,3]> with collagen yields of 1% or greater [33].

		this study						Sümegi *et al.* (2012)		
**Species**	**Locality (layer)**	**Collagen (%)**	**C/N ratio**	**C-14 age (BP)**	**±**	**Calendar age 1σ (cal BP)**	**Calendar age 2σ (cal BP)**	**C-14 age (BP)**	**±**	**deviation**
Sus scrofa	Rejtek. Block II. 90-120 cm (8)	14.8	3.5	8768	50	9558-9927	9685-9890			
Clethrionomys glareolus	Rejtek. Block II. 90-120 cm (8)	12.2	3.6	6959	49	7684-7871	7720-7843	4675	35	2284
Ochotona sp.	Rejtek. Block III. 140-160 cm (9)	12.2	3.5	10243	65	11714-12238	11915-12090			
Meles meles	Rejtek. Block III. 140-160 cm (9)	20.9	3.5	9727	50	11077-11242	11129-11219			
Arvicola terrestris	Rejtek. Block III. 140-160 cm (9)	10.8	3.6	9594	53	10746-11155	10788-10973	8970	50	624
Arvicola terrestris	Rejtek. Block III. 160-180 cm (10)	13.0	3.5	10392	62	12028-12434	12146-12397			
Cricetus cricetus	Rejtek. Block III. 160-180 cm (10)	12.5	3.4	10125	60	11591-12016	11616-11840	9790	40	335
Lagopus lagopus	Rejtek. Block III. 160-180 cm (10)	18.9	3.4	12527	76	14607-15060	14306-15120			
Microtus arvalis	Rejtek. Block III. 180-200 cm (11)	11.1	3.4	11527	71	13206-13489	13302-13433			
Cricetus cricetus	Rejtek. Block III. 180-200 cm (11)	13.5	3.4	10342	61	11952-12420	12063-12240			
Lagopus lagopus	Rejtek. Block III. 180-200 cm (11)	10.5	3.5	12575	73	14427-15192	14745-15098	12260	50	315
Microtus arvalis	Rejtek. Block III. 220-220 cm (12)	11.6	1.7*	11792	76	13466-13757	13553-13725	12530	50	-738
Microtinae	Rejtek. Block III. 220-220 cm (12)	11.6	3.3	11676	71	13349-13643	13439-13566			
Lasiopodomys (Stenocranius) gregalis	Rejtek. Block III. 220-220 cm (12)	10.5	3.4	11496	72	13181-13472	13278-13420			
Ochotona sp.	Jankovich Cave, Block II. 110-130 cm (5)	6.6	3.3	12867	73	15216 - 15489	15130 - 15638			
Rangifer tarandus	Jankovich Cave, Block II. 110-130 cm (5)	3.7	3.4	12753	77	15084 - 15309	14885 - 15496			
Spermophilus sp.	Jankovich Cave, Block II. 130-160 cm (6)	5.1	3.4	13106	74	15590 - 15872	15401 - 15992			
Dicrostonyx sp.	Jankovich Cave, Block II. 130-160 cm (6)	5.5	NA	13765	95	16457 - 16821	16314 - 16973			
Ochotona sp.	Jankovich Cave, Block II. 150-170 cm (7)	6.8	3.3	13535	79	16165 - 16430	16047 - 16588			
Ochotona sp.	Jankovich Cave, Block II. 170-200 cm (8)	6.8	3.3	13677	78	16334 - 16630	16232 - 16813			
Ochotona sp.	Jankovich Cave, Block II. 200-220 cm (9)	6.6	3.3	13967	85	16790 - 17106	16594 - 17243			
Dicrostonyx sp.	Jankovich Cave, Block II. 220-250 cm (10)	5.9	3.4	14454	90	17483 - 17749	17363 - 17897			
Ochotona sp.	Jankovich Cave, Block II. 220-250 cm (10)	7.6	3.3	14378	90	17389 - 17658	17224 - 17814			

*cautious

Supplementary Table 4 Radiocarbon dates obtained directly on woolly mammoth (*Mammuthus primigenius*) bones or on associated charcoal or mollusc remains at different sites in the Carpathian Basin. Sites for this study were selected to reflect the possibly youngest specimens in the Carpathian Basin. Bones presented in this study first were sampled in the mammoth bone collection of the Hungarian Natural History Museum, The Mátra Museum and the Tiszaföldvár Geographical Museum. Recommended values for C/N ratios of bulk bone or tooth range from 2.9 to 3. [31,3]> with collagen yields of 1% or greater [33].

Location	Sample	Laboratory code	Collagen (%)	C/N ratio	^{14}C age (BP)	Calendar age 2σ (cal BP)	Reference	Inventory number (NHMUS)
Csajág	tooth	GdA-2011	-	-	13315±35	15830-16185	Katona et al. (2010); Kovács et al. (2012)[34,3]>	-
Pilismarót - Clay Pit	tooth	DeA-13790	4.4	3.5	14845±98	17820-18330	this study	V.60.630.
Feldebrő[1]	femur⸸	DeA-13792	1.7	3.5	15314±103	18470-18880	this study	uninventoried
Feldebrő[1]	femur⸸	DeA-13793	1.6	3.5	15168±104	18140-18680	this study	uninventoried
Szurdokpüspöki	rib	DeA-11625	3.7	3.4	15866±113	18880-19470	this study	uninventoried
Budapest-Csillaghegy	mollusc[2]	Deb-3160	-	-	15935 ± 150	18880-19590	Sümegi and Hertelendi (1998)[3]>	-
Szeged-Öthalom	bone⸸	Deb-3344	-	-	15916±168	18830-19605	Sümegi and Krolopp (1995)[3]>	-
Esztergom - Gyurgyalag	charcoal⸸	Deb-1160	-	-	16160±200	18195-19995	Hertelendi (1991)[3]>	-
Tápiósüly	charcoal[3]	Hv-1615	-	-	16750±400	19210-21235	Geyh et al. (1969)[3]>	-
Zók	tusk[4]	AA-80678	-	-	17760±200	20905-21995	Konrád et al. (2010)[4]>	-
Zók	tusk[4]	Deb-14677	-	-	20500±1500[5]	21174-27703	Konrád et al. (2010)[4]>	-
Mátraderecske	tooth	DeA-13760	3.9	3.2	20798±194	24490-25560	this study	V.72.37.
Tarcal - Brickyard	tooth	DeA-13788	3.9	3.4	21106±193	24970-25860	this study	uninventoried
Bodrogkeresztúr	tooth	DeA-13758	12.5	3.3	21275±188	25200-25940	this study	V.60.634.
Petőfibánya Gravel Pit	tooth	DeA-14807	2.4	NA	22200±230	25970-27040	this study	80.9.1.
Feldebrő	tooth	DeA-14806	14.3	NA	22300±170	26090-27070	this study	78.9.
Bodrogkeresztúr-Henye	femur⸸	Poz-95146	-	-	22620±210	26416-27401	Wilczyński et al. (2020)[4]>	-
Tarcal – Brickyard	tooth	DeA-13759	5.9	3.4	22599±217	26360-27390	this study	uninventoried
Mende	charcoal	Mo-422	-	-	29800±600	32485-35005	Vinogradov et al. (1968)[4]>	-
Tata – Porhanyó Quarry	charred bone⸸[6]	GrN-3023	-	-	33330±900	35490-39645	Vogel and Waterbolk (1967)[4]>	-
Kiskunlacháza	tooth	DeA-13791	1.1	3.9	37524±1103	39700-43480	this study	V.72.90.
Tisza river	mandible	DeA-21340	3.2		39200±1000	42225-43410	this study	86.59.
Kiskunlacháza	tooth	DeA-13765	3.8	3.4	40000 <		this study	V.80.119.
Ócsa-Felsőbabád	tooth	DeA-13789	5.7	3.3	40000 <		this study	V.80.120.
Lágymányos	tooth	DeA-13766	5.1	3.5	40000 <		this study	V.72.15.
Gyoma. Tisza-meder	mandible	DeA-13784	6.7	3.6	40000 <		this study	V.79.29.
Tisza river	tooth	DeA-13782	4.6	3.5	40000 <		this study	V.63.25.
Törökbecse	mandible	DeA-13783	5.0	3.7	40000 <		this study	V.79.24.
Tisza river	mandible	DeA-11626	10.9	3.6	40000 <		this study	V.63.44.
Tisza river	mandible	DeA-13761	11.6	3.6	40000 <		this study	V.63.3.
Zebegény	tooth	DeA-13787	4.6	3.4	40000 <		this study	V.79.207.
Tisza river	tooth	DeA-21330	8,4		40000 <		this study	86.6.
Tisza river	tooth	DeA-21332	7,8		40000 <		this study	86.7.
Tisza river	tooth	DeA-21335	14,3		40000 <		this study	86.16.

Tószeg, Tisza river	tooth	DeA-21337	6,7		40000 <		this study	86.36.
Tisza river	mandible	DeA-21339	6,1		40000 <		this study	86.64.
Zalaegerszeg, 2nd Brick Factory	tooth	DeA-21341	3,6		40000 <		this study	55.42.1.
Zalaszentgrót (construction)	tooth	DeA-21342	8,4		40000 <		this study	57.93.1.
Dunavarsány	tooth	na	<1%	-	-		this study	V.88.10.
Felsőzsolca	tooth	na	<1%	-	-		this study	V.63.1344.
Püspökhatvan	tusk	na	<1%	-	-		this study	V.72.126.
Tisza river	tooth	na	<1%	-	-		this study	V.63.56.
Heves – Gravel Pit	tooth	na	<1%	-	-		this study	78.22.1.
Andráshida, Apátfalvi Gravel Pit	tooth	na	<1%	-	-		this study	57.94.1.
Tisza river	tooth	na	<1%	-	-		this study	86.11.1.
missing locality	tooth	na	<1%	-	-		this study	86.49.
missing locality	tooth	na	<1%	-	-		this study	59.314.1
Zalaszentmihályfa (Alsómajor)	tooth	na	<1%	-	-		this study	uninventoried
Pilismarót, Andráshida	tooth	na	<1%	-	-		this study	50.01.2.
Zalaegerszeg, 1st Brick Factory	tooth	na	<1%	-	-		this study	57.92.1.

⸸in archaeological context
[1]same locality
[2]located *ca*. 0.2 m under mammoth remain in archaeological layer
[3] located ca. 3.0 m above mammoth remain
[4]same tusk
[5]conventional ^{14}C dating
[6]from archaeological layer sandwiched by travertine beds dated to *ca*. 100 ka

Supplementary Table 5 Radiocarbon dates obtained either directly on reindeer (*Rangifer tarandus*) bones or charcoal pieces from the same sediment layer at different archaeological sites in the Carpathian Basin. Sites were selected to reflect the youngest dated specimens/sites in the Carpathian Basin. Note that earlier ^{14}C dates from Jankovich Cave published in Pazonyi[4]> were obtained from unidentified mammal bone fragments.

Site	Dated material	^{14}C age	Calendar age 2σ (98%) (cal BP)	Reference
Jankovich Cave	bone	11720 ± 190	13170-14000	Pazonyi (2006)[4]>
Jankovich Cave	bone	12440 ± 230	13810-15310	Pazonyi (2006)[4]>
Zöld Cave layer 3	charcoal	12702±55	14974-15304	Béres et al. (2021)[4]>
Jankovich Cave, Block II. 110-130 cm (layer 5)	bone	12753±77	14885-15495	this study
Zöld Cave layer 3	bone	13110±90	15415-16008	Béres et al. (2021)[4]>
Jankovich Cave, Block I. 20-40 cm	bone	14613±88	17555-18015	this study
Arka	charcoal	17050 ± 350	19700-21505	Vogel and Waterbolk (1964)[4]>
Jankovich Cave, Block II. 90-110 cm (layer 4)	bone	16894±123	20045-20685	this study
Ságvár	charcoal	17760 ± 350	20620-22340	Vogel and Waterbolk (1964)[4]>
Jászfelsőszentgyörgy	bone	18500 ± 400	21380-23340	Hertelendi (1993)[4]>
Herman Ottó Cave layer 5	bone	18480±90	22198-22585	Szolyák (2009)[4]>
Herman Ottó Cave layer 5	bone	18650 ± 90	22385-22885	Szolyák (2009)[4]>
Ságvár	charcoal	18900 ± 100	22490-23025	Vogel and Waterbolk (1964)[4]>
Herman Ottó Cave layer 5	bone	24290±140	27995-28820	Szolyák (2009)[4]>
Pilisszántó I Rockshelter lower layer	bone	24990±270	28740-29920	Wilczyński et al. (2020)[4]>
Pilisszántó I Rockshelter lower layer	bone	25150±280	28865-30000	Wilczyński et al. (2020)[4]>
Peskő Cave	bone	35200 ± 670	38425-41230	Vogel and Waterbolk (1972)[5]>

Supplementary Figure 1 Location of Jankovich Cave in Hungary and sketch map of the cave interior with the location of the excavation blocks. The map in the top left corner is a satellite image from ArcGIS (basemaps). The software used to create the map was ArcGIS 10.2.2 for desktop, version 10.2.2.3552. Software url: https://support.esri.com/en/products/desktop/arcgis-desktop/arcmap/10-2-2. The photo of Jankovich Cave was taken by Mihály Gasparik.

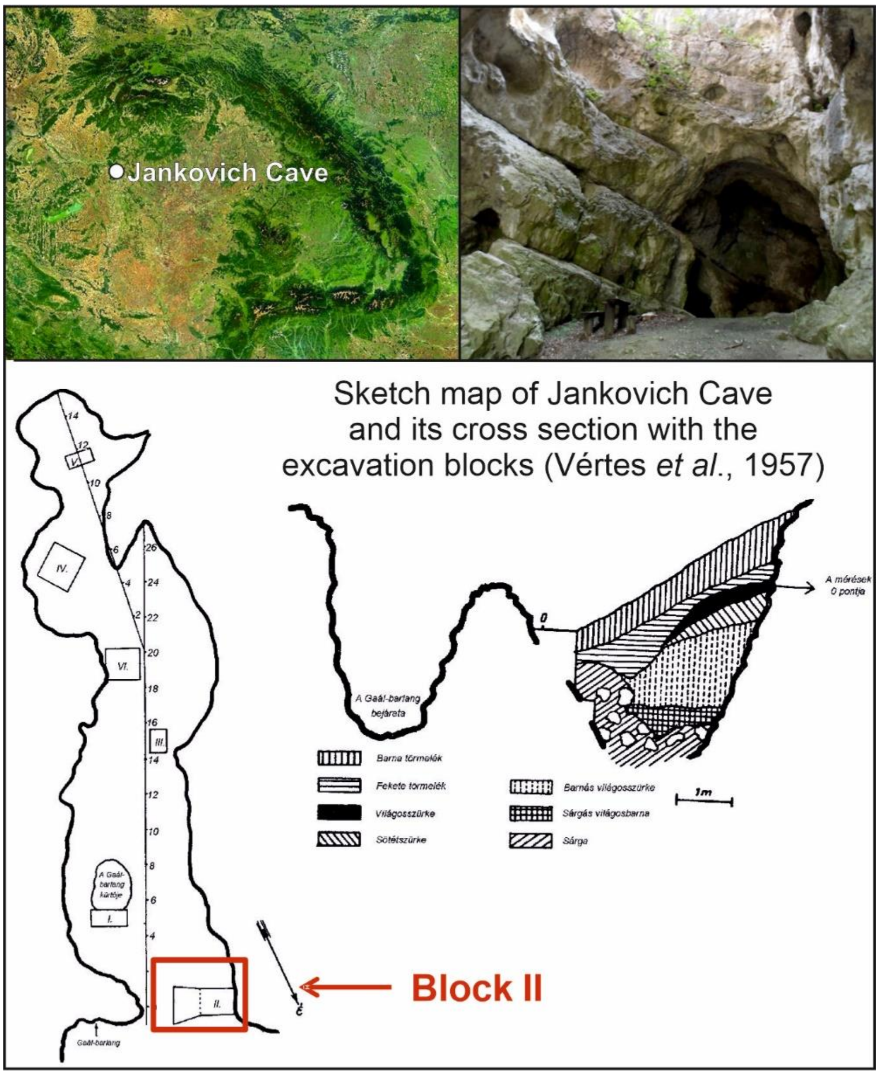

Supplementary Figure 2 Location of Rejtek I. Rock Shelter in Hungary and sketch map of the cave interior with the location of the excavation blocks. The map in the top left corner is a satellite image from ArcGIS (basemaps). The software used to create the map was ArcGIS 10.2.2 for desktop, version 10.2.2.3552. Software url: https://support.esri.com/en/products/desktop/arcgis-desktop/arcmap/10-2-2. The photo of Rejtek I Rock Shelter was taken by Tivadar Czina under licence CC BY-SA 3.0, source: https://hu.wikipedia.org/wiki/Rejteki_1._sz._kőfülke

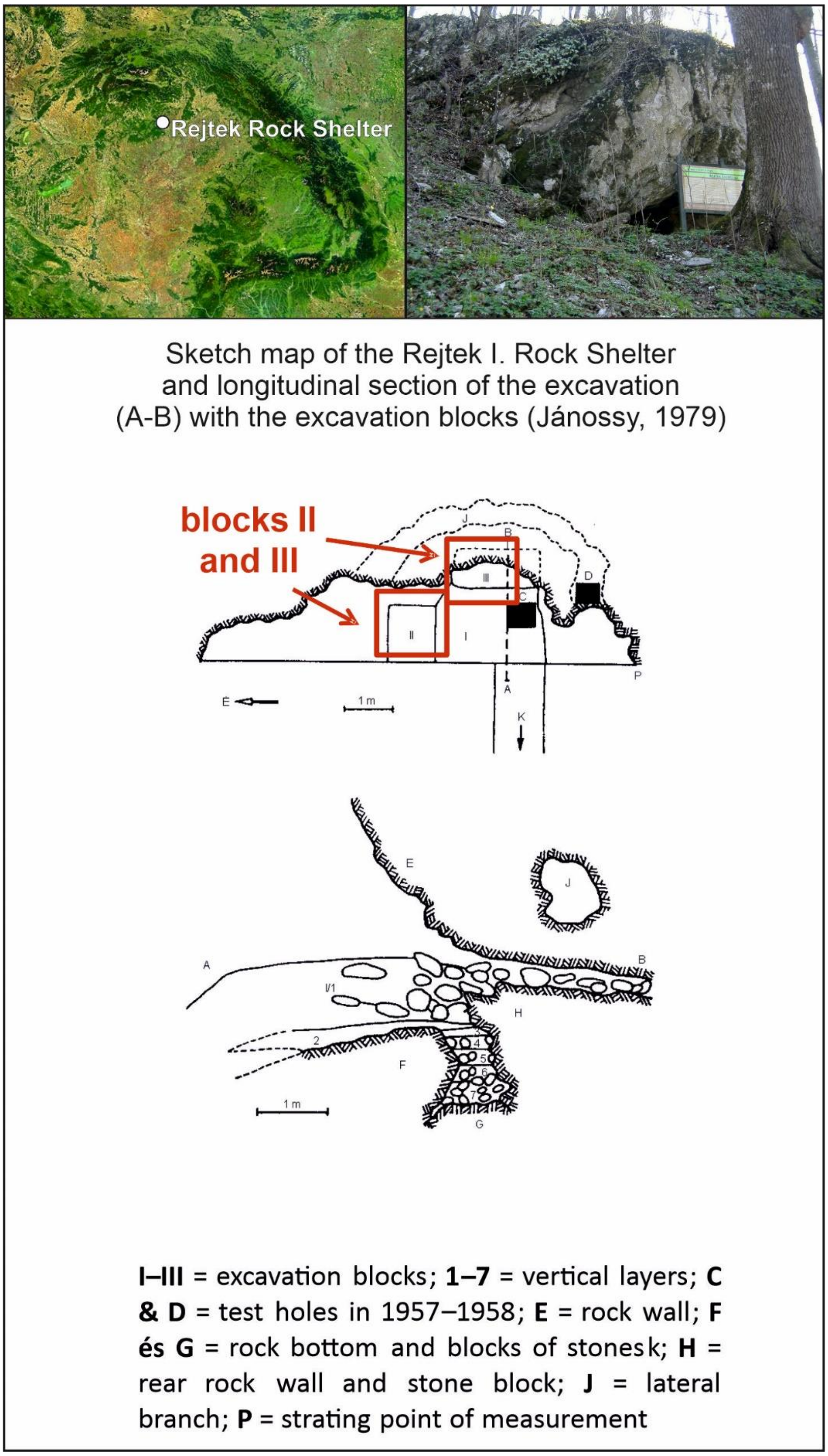

Supplementary Figure 3 Micromammal and tree vegetation compositional changes along the Rejtek I. Rock shelter sediment profile based on bone assemblage and charcoal studies[5,6,5]>.

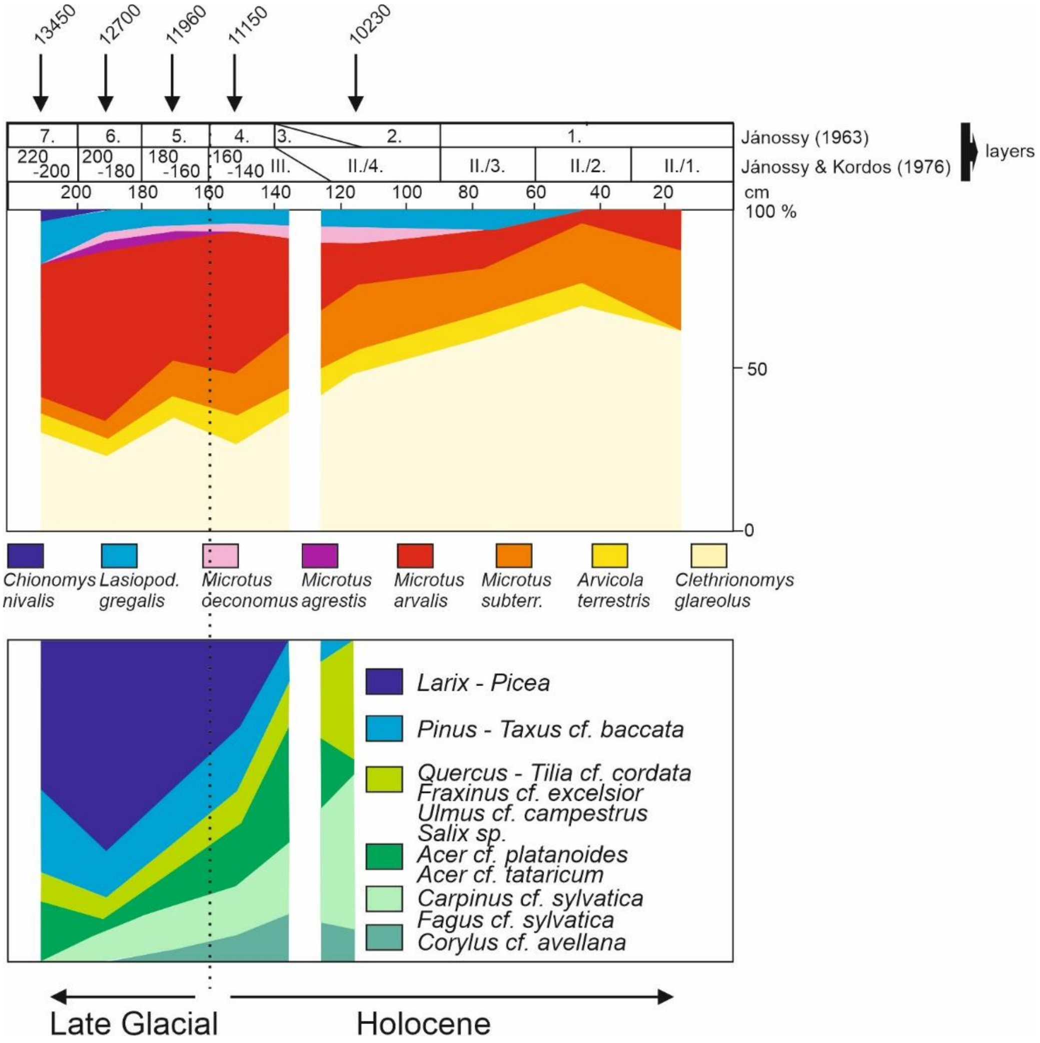

Supplementary Figure 4 Bayesian age-depth models from Rejtek I. Rock Shelter and Jankovich Cave based on radiocarbon data in Suppl. Table 1.

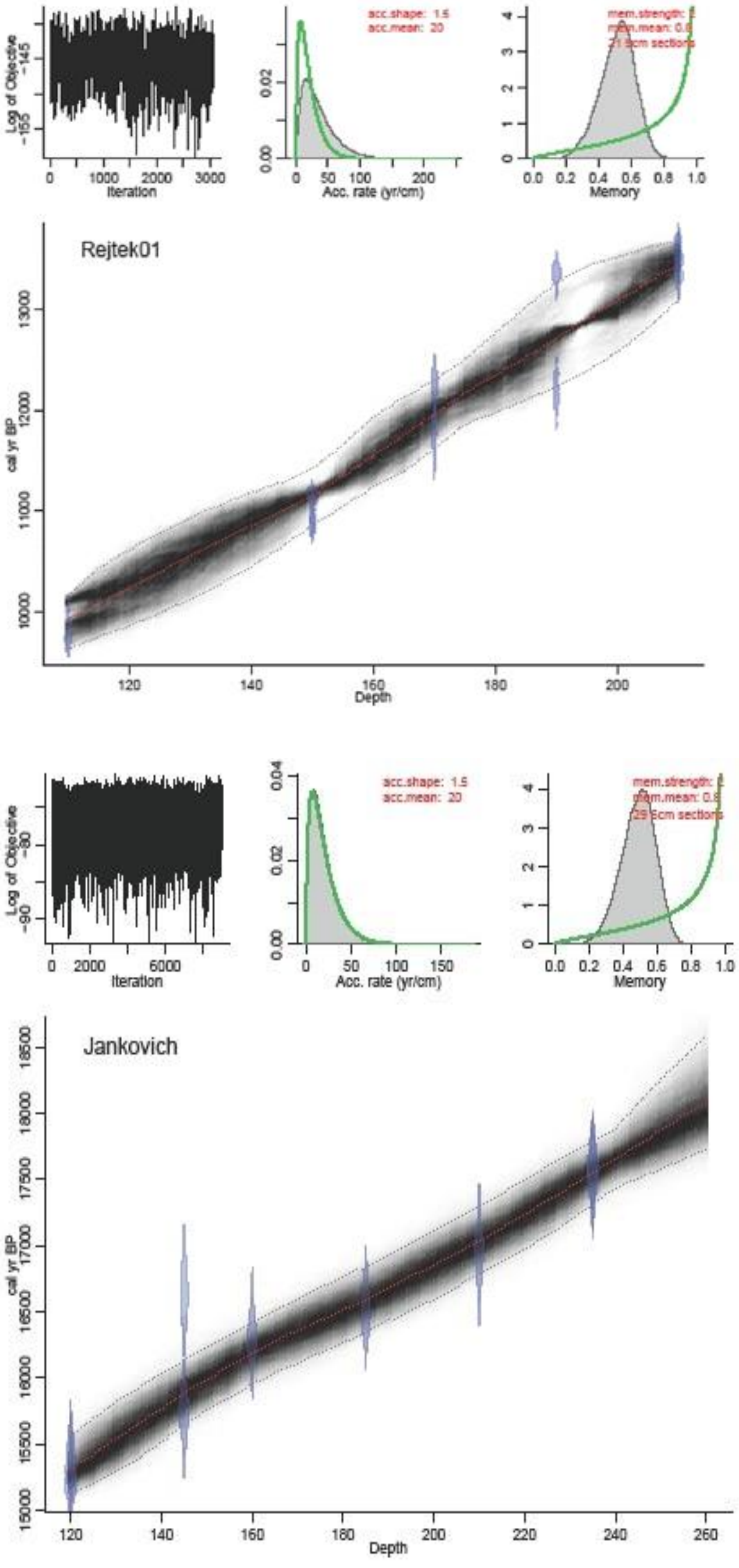

Supplementary Figure 5 Vole community changes (relative frequencies) in Jankovich Cave plotted against age. On the right side of the figure δ18O record of the NGRIP ice core is shown along the GICC05 age model and ice core event stratigraphy[52]; YD: Younger Dryas; HE-1: Heinrich-event 1

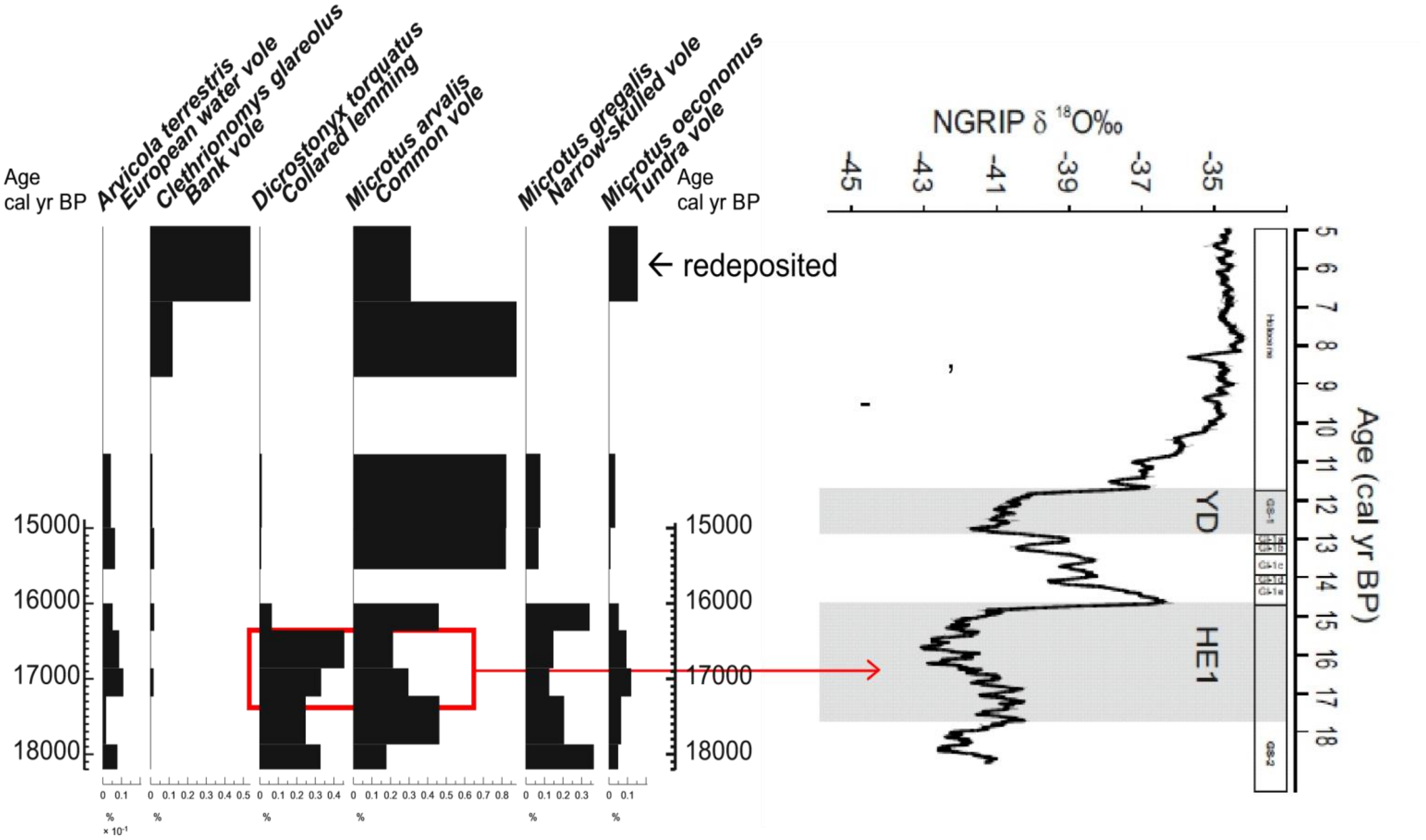

Supplementary Figure 6 Vole and rodent community changes (relative frequencies) in Rejtek I. Rock Shelter plotted against age.

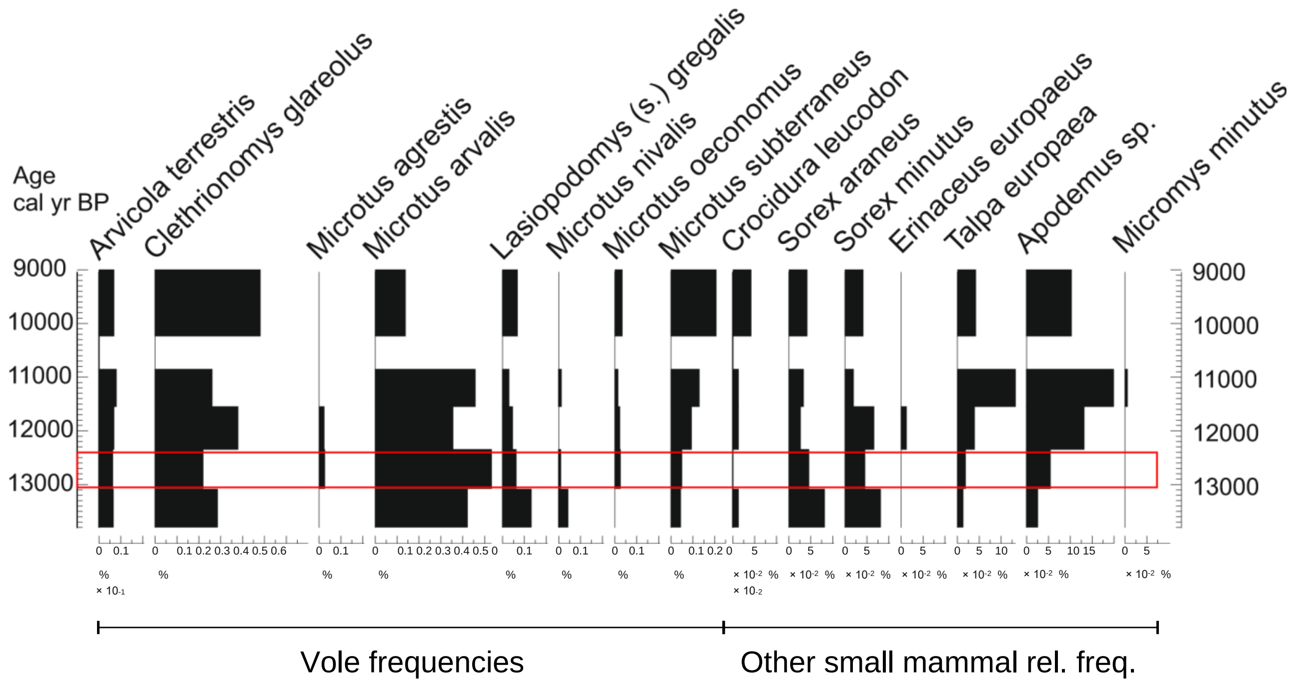

Supplementary Figure 7 2σ calibrated age ranges of Late Pleniglacial reindeer (*Rangifer tarandus*) bones and associated charcoal material from archaeological sites in the Carpathian Basin based on data published in Suppl. Table 5; median ages are presented as horizontal red squares, the lilac bar represents OxCal Phase estimate for the last appearance of reindeer

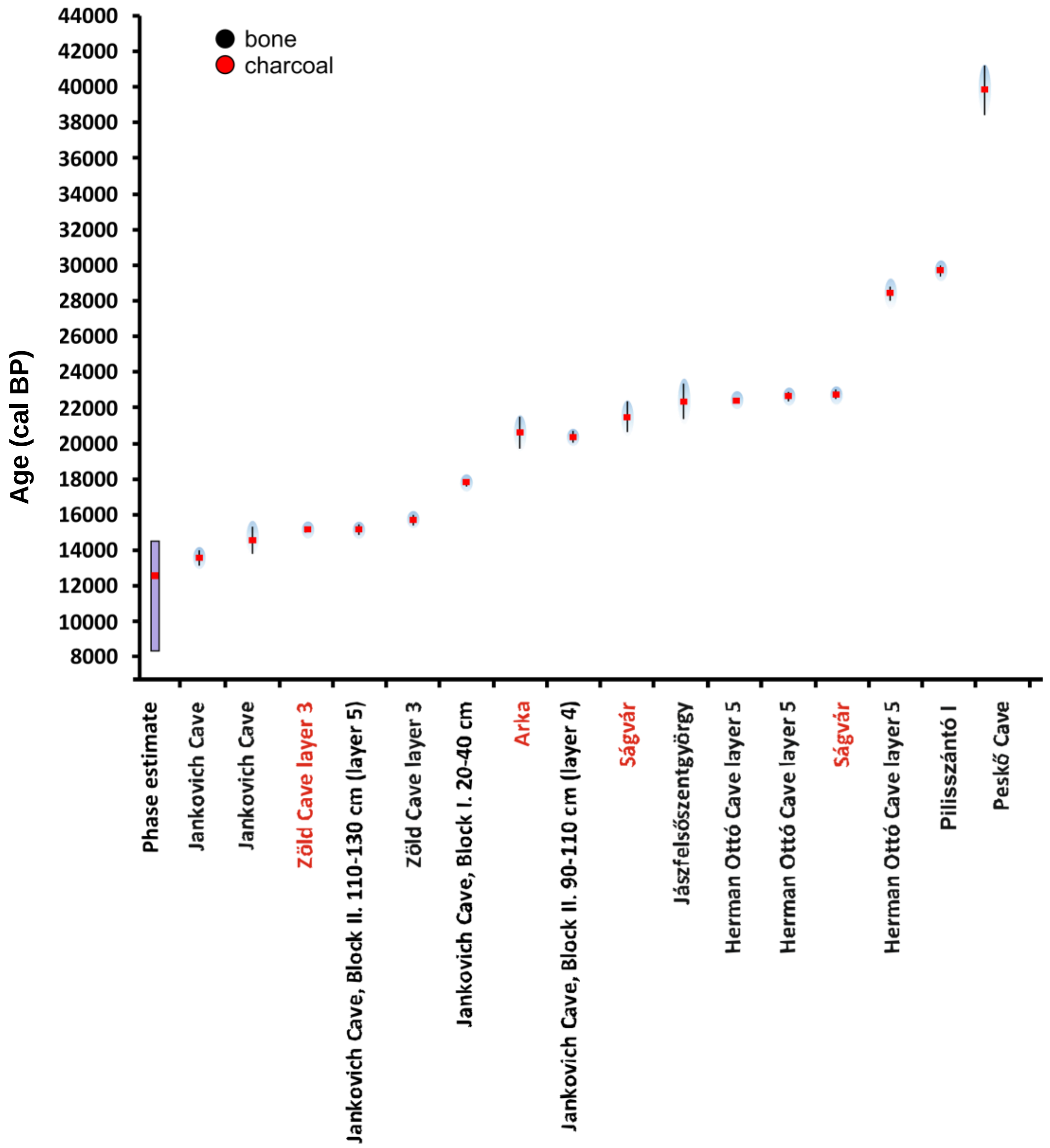

Supplementary Figure 8 2σ calibrated age ranges of woolly mammoth (*Mammuthus primigenius*) bones and associated charcoal material from archaeological sites in the Carpathian Basin based on data published in Suppl. Table 4; median ages are presented as horizontal red squares, the lilac bar represents OxCal Phase estimate for the last appearance of woolly mammoth; the lower figure shows the δ^{18}O record of the NGRIP ice core along the GICC05 age model[5]> with grey shaded rectangles highlighting periods of apparent woolly mammoth absence in the Carpathian Basin.

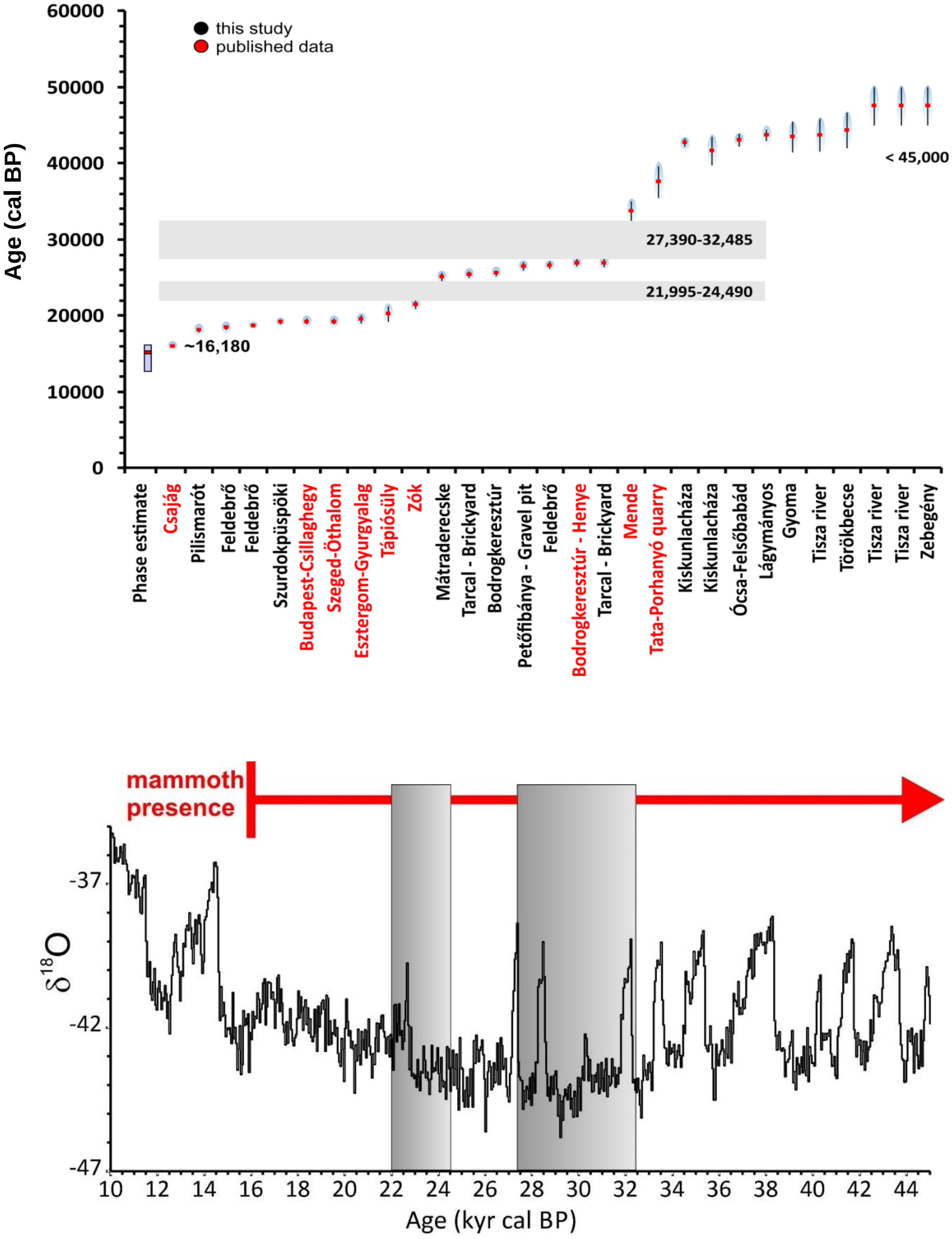

Supplementary Figure 9 Kokad mire diversity, richness and evenness indicators calculated in the R Vegan package using modified scripts of Giesecke et al.[5]>; a) Sample diversity measures, N0: palynological richness E(T317); N1: Hill number or effective number of species, exponential of the Shannon entropy calculated with natural logarithms; N2: effective diversity, inverse of the Simpson diversity; b) N2/N0; c) evenness as the inverse slope of the rank-order abundance of taxa exceeding 1 % in the sample; d) number of pollen types in three consecutive samples as a running three-term-taxa-accumulation from the base to the top of the diagram; e) taxa accumulation curve**.**

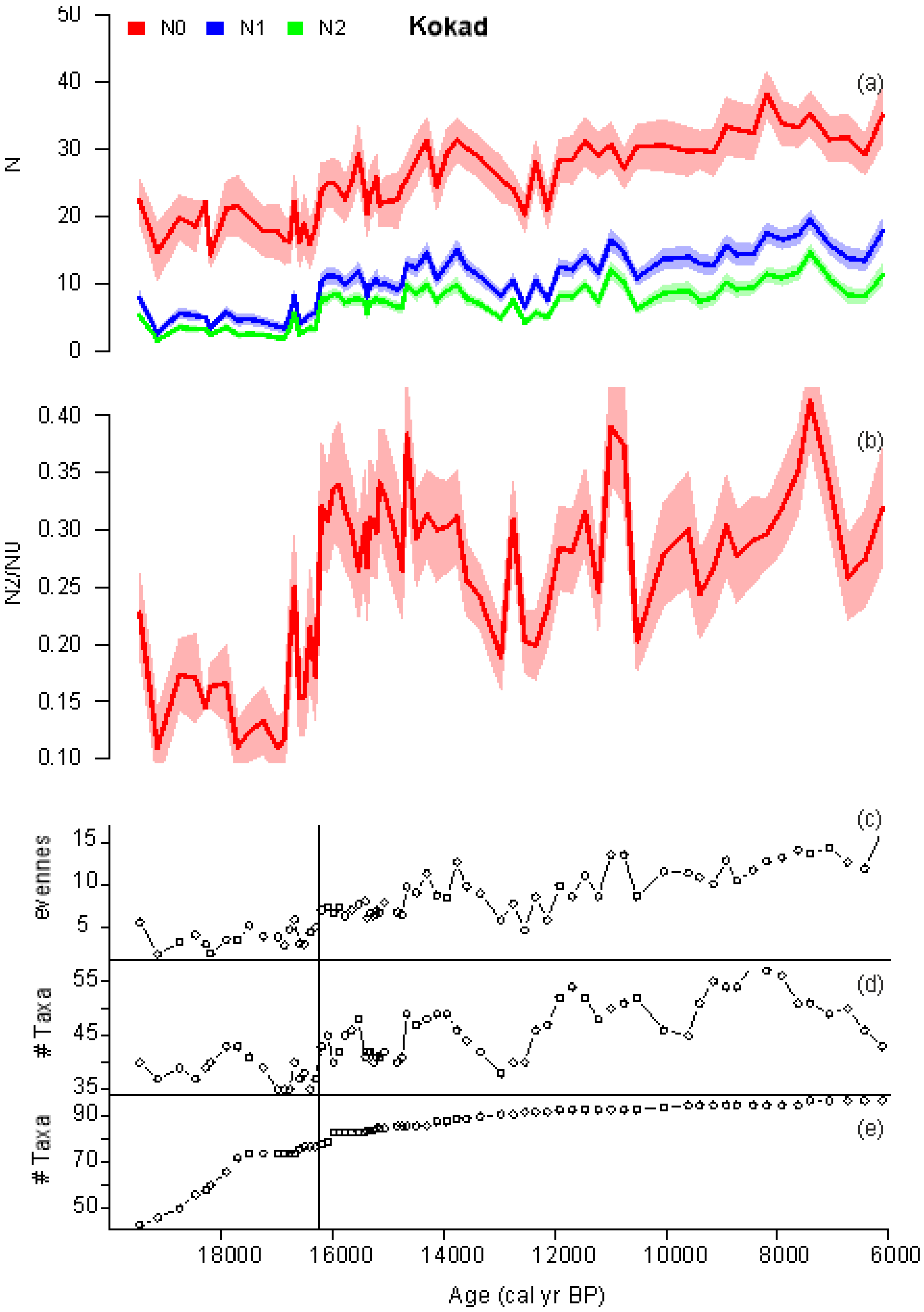

Supplementary Figure 10 Lake St Anne diversity, richness and evenness indicators calculated in the R Vegan package using modified scripts of Giesecke et al.[5]>; a) Sample diversity measures, N0: palynological richness E(T317); N1: Hill number or effective number of species, exponential of the Shannon entropy calculated with natural logarithms; N2: effective diversity, inverse of the Simpson diversity; b) N2/N0; c) evenness as the inverse slope of the rank-order abundance of taxa exceeding 1 % in the sample; d) number of pollen types in three consecutive samples as a running three-term-taxa-accumulation from the base to the top of the diagram; e) taxa accumulation curve.

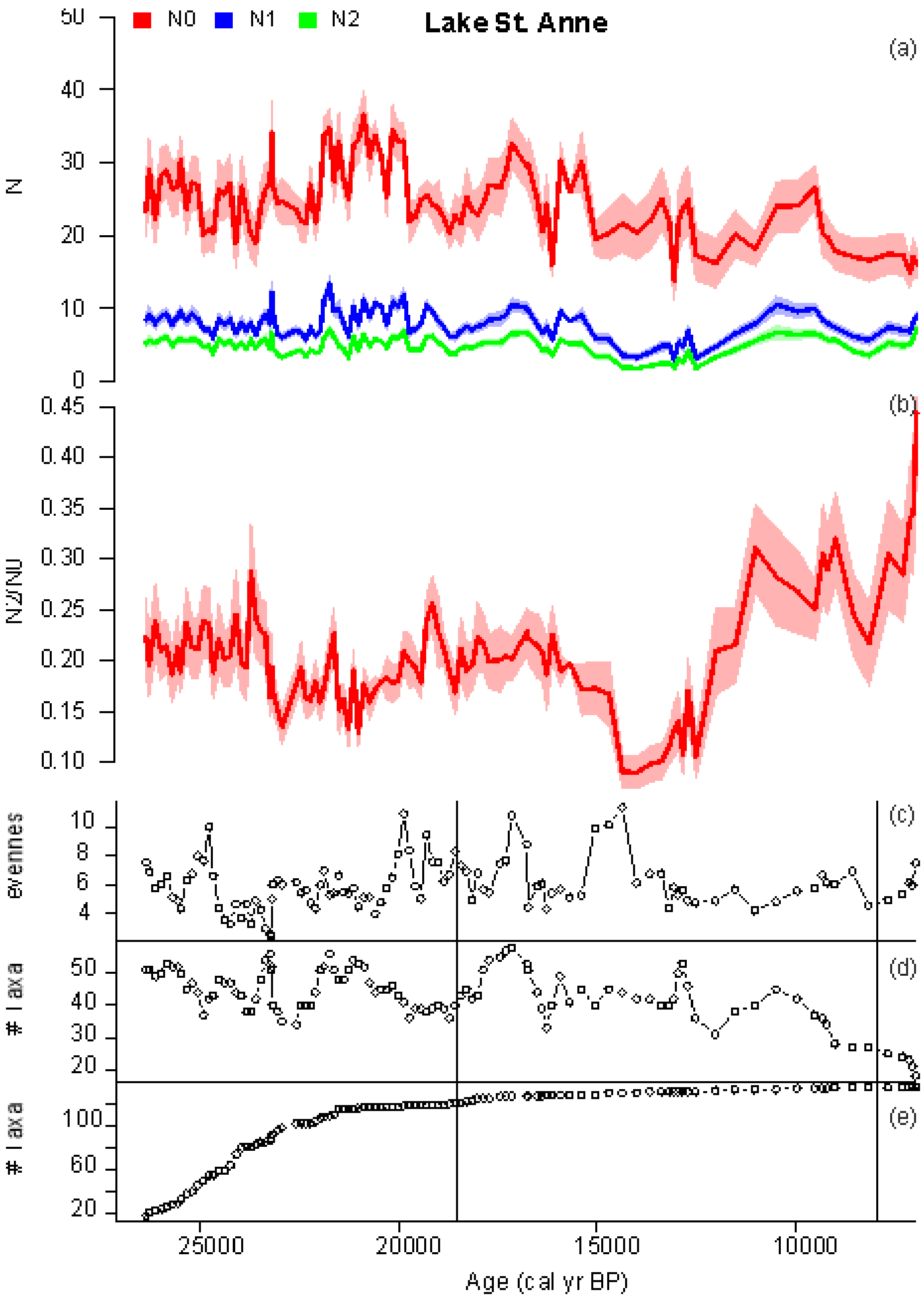

Supplementary Figure 11 Regime shift analysis using principal component 1st axis scores from the Kokad Mire pollen record as an estimate of terrestrial vegetation change[24] and Bayesian change point analysis (multi change point analysis (MCP) in R)[5]>. Visual inspection of the stratigraphic PC plot suggested the presence of minimum 2 change points, therefore this a priori information was used in the model. Latest ^{14}C dates and Phase estimates of extinction times are shown in addition to the change point to examine the order of events. Yellow background represents the 2σ calibrated age ranges of the latest ^{14}C dates.

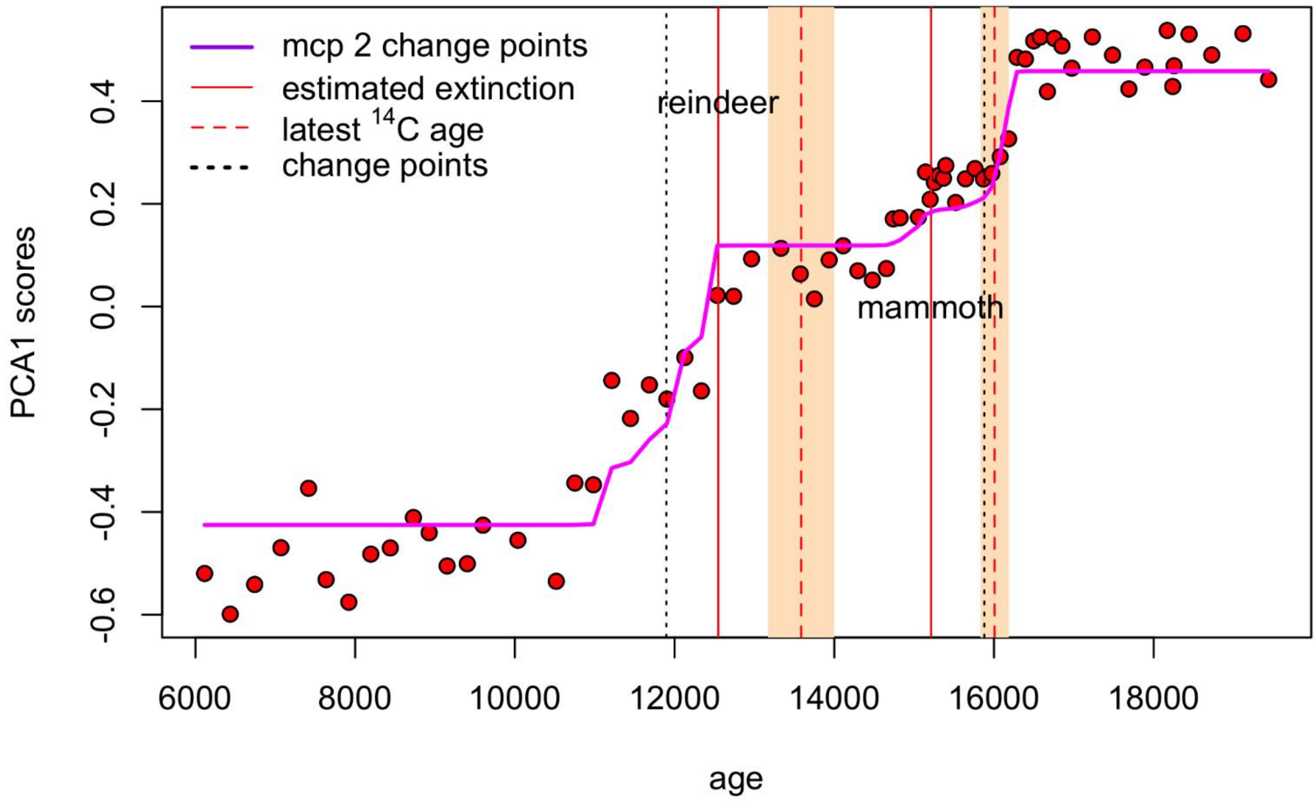

References

1. Vértes, L. Neuere Forschungen in der Jankovich-Höhle. *Folia Archaeol.* **9**, 3–16 (1957).
2. Hillebrand, J. Results of my cave research in 1916 (Az 1916. évi barlangkutatásaim eredményéről). *Barlangkutatás* **5**, 98–108 (1917).
3. Lambrecht, K. & Kormos, T. Die Fauna der Öregköhöhle bei Bajót (A bajóti Öregkő nagy barlangjának faunája). *Barlangkutatás* **2**, 77–80 (1914).
4. Vértes, L., Kretzoi, M. & Herrmann, M. Neuere Forschungen in der Jankovich-Höhle. *Folia Archaeol.* **9**, 3–23 (1957).
5. Jánossy, D. & Kordos, L. Pleistocene-Holocene Mollusc and Vertebrate Fauna of two caves in Hungary. *Ann. Hist. Musei Natl. Hungarici* **68**, 5–29 (1976).
6. Jánossy, D. Vorläufige Ergebnisse der Ausgrabungen in der Felsnische Rejtek I. (Bükkgebirge, Gem. Répáshuta). *Karszt- és Barlangkutatás* **3**, 49–58 (1961).
7. Stieber, J. Esami antracotomici sulla storia della vegetazione tardiglaciale in Ungheria (A hazai későglaciális vegetációtörténet anthrakotómiai vizsgálatok alapján). *Földtani Közlöny* **99**, 188–193 (1969).
8. Sümegi, P., Rudner, E. & Törőcsik, T. Environmental and chronological reconstruction problems during the Pleistocene/Holocene transition in Hungary (Magyarország pleisztocén végi és kora holocén környezeti változások kronológiai, tér és időbeli rekonstrukciós problémái). in *Őskoros kutatók IV. összejövetelének konferenciakötete* (ed. Kolozsi, B.) 279–298 (Hajdú-Bihar Megyei Múzeumok Igazgatósága, 2012).
9. Vértes, L. *Paleolithic and Mesolithic remains in Hungary (Az őskőkor és az átmeneti kőkor emlékei Magyarországon)*. (Akadémiai Kiadó, 1965).
10. Jánossy, D. *Pleistocene vertebrate faunas of Hungary*. *Journal of Chemical Information and Modeling* (Akadémiai Kiadó, 1986).
11. Magyari, E. *et al.* Radiocarbon chronology of glacial lake sediments in the Retezat Mts (South Carpathians, Romania): A window to Late Glacial and Holocene climatic and paleoenvironmental changes. *Cent. Eur. Geol.* **52**, 225–248 (2009).
12. Magyari, E. K. *et al.* Rapid vegetation response to Lateglacial and early Holocene climatic fluctuation in the South Carpathian Mountains (Romania). *Quat. Sci. Rev.* **35**, 116–130 (2012).
13. Korponai, J. *et al.* Cladocera response to Late Glacial to Early Holocene climate change in a South Carpathian mountain lake. *Hydrobiologia* **676**, 223–235 (2011).
14. Tóth, M. *et al.* A chironomid-based reconstruction of late glacial summer temperatures in the southern Carpathians (Romania). *Quat. Res.* **77**, 122–131 (2012).
15. Buczkó, K. *et al.* Responses of diatoms to the Younger Dryas climatic reversal in a South Carpathian mountain lake (Romania). *J. Paleolimnol.* **48**, 417–431 (2012).
16. Braun, M. *et al.* Using linear discriminant analysis (LDA) of bulk lake sediment geochemical data to reconstruct lateglacial climate changes in the South Carpathian Mountains. *Quat. Int.* **293**, 114–122 (2013).
17. Hubay, K. *et al.* Reconstruction of paleoenvironmental changes using geochemical data from South Carpathian Mountains. *EGU2020-13551* (2020).
18. Vincze, I. *et al.* Paleoclimate reconstruction and mire development in the Eastern Great Hungarian Plain for the last 20,000 years. *Rev. Palaeobot. Palynol.* **271**, 104112 (2019).
19. Karátson, D. *et al.* The latest explosive eruptions of Ciomadul (Csomád) volcano, East Carpathians - A tephrostratigraphic approach for the 51-29 ka BP time interval. *J. Volcanol. Geotherm. Res.* **319**, 29–51 (2016).

20. Magyari, E. K. *et al.* Vegetation and environmental responses to climate forcing during the Last Glacial Maximum and deglaciation in the East Carpathians: Attenuated response to maximum cooling and increased biomass burning. *Quat. Sci. Rev.* **106**, 278–298 (2014).

21. Magyari, E. K. *et al.* Late Pleniglacial vegetation in eastern-central Europe: Are there modern analogues in Siberia? *Quat. Sci. Rev.* **95**, 60–79 (2014).

22. Magyari, E., Jakab, G., Rudner, E. & Sümegi, P. Palynological and plant macrofossil data on Late Pleistocene short-term climatic oscillations in NE-Hungary. *Acta Palaeobot. Suppl.* **2**, 491–502 (1999).

23. Magyari, E., Sümegi, P., Braun, M., Jakab, G. & Molnár, M. Retarded wetland succession: Anthropogenic and climatic signals in a holocene peat bog profile from north-east hungary. *J. Ecol.* **89**, 1019–1032 (2001).

24. Magyari, E. K. *et al.* Warm Younger Dryas summers and early late glacial spread of temperate deciduous trees in the Pannonian Basin during the last glacial termination (20-9 kyr cal BP). *Quat. Sci. Rev.* **225**, (2019).

25. Hubay, K. Dating of pollen samples from the sediment core of Lake St Anne in the East Carpathian Mountains , Romania. *Geophys. Res. Abstr.* **18**, 12260 (2016).

26. Higuera, P. E., Gavin, D. G., Bartlein, P. J. & Hallett, D. J. Peak detection in sediment-charcoal records: Impacts of alternative data analysis methods on fire-history interpretations. *Int. J. Wildl. Fire* **19**, 996–1014 (2010).

27. Ramsey, C. B. & Lee, S. Recent and Planned Developments of the Program OxCal. *Radiocarbon* **55**, 720–730 (2013).

28. Kosintsev, P. *et al.* Evolution and extinction of the giant rhinoceros Elasmotherium sibiricum sheds light on late Quaternary megafaunal extinctions. *Nat. Ecol. Evol.* **3**, 31–38 (2019).

29. McInerny, G. J., Roberts, D. L., Davy, A. J. & Cribb, P. J. Significance of sighting rate in inferring extinction and threat. *Conserv. Biol.* **20**, 562–567 (2006).

30. Bradshaw, C. J. A., Cooper, A., Turney, C. S. M. & Brook, B. W. Robust estimates of extinction time in the geological record. *Quat. Sci. Rev.* **33**, 14–19 (2012).

31. Ramsey, C. B., Higham, T., Bowles, A. & Hedges, R. Improvements to the pretreatment of bone at Oxford. *Radiocarbon* **46**, 155–163 (2004).

32. van der Plicht, J. & Palstra, S. W. L. Radiocarbon and mammoth bones: What's in a date. *Quat. Int.* **406**, 246–251 (2016).

33. Van Klinken, G. J. Bone collagen quality indicators for palaeodietary and radiocarbon measurements. *J. Archaeol. Sci.* **26**, 687–695 (1999).

34. Katona, L., Kovács, J., Kordos, L., Linkai, I. & Magyari, Á. Two young woolly mammoths findings near Lake Balaton, Hungary. *Quat. Hors-série 3* 75–77 (2010).

35. Kovács, J., Moravcová, M., Újvári, G. & Pintér, A. G. Reconstructing the paleoenvironment of East Central Europe in the Late Pleistocene using the oxygen and carbon isotopic signal of tooth in large mammal remains. *Quat. Int.* **276**–**277**, 145–154 (2012).

36. Sümegi, P. & Hertelendi, E. Reconstruction of microenvironmental changes in the Kopasz Hill loess area at Tokaj (Hungary) between 15 and 70 ka BP. *Radiocarbon* **40**, 855–863 (1998).

37. Sümegi, P. & Krolopp, E. Reconstruction of palaeoecological conditions during the deposition of Würm loess formations of Hungary, based on molluscs (A magyarországi würm korú löszök képződésének paleoökológiai rekonstrukciója Mollusca-fauna alapján). *Földtani Közlöny* **125**, 125–148 (1995).

38. Hertelendi, E. Radiocarbon dating of a wood sampl e from an excavation near Esztergom-Gyurgyalag. *Acta Archaeol. Acad. Sci. Hungaricae* **43**, 271–272 (1991).

39. Geyh, M. A., Schweitzer, F., Vértes, L. & Vogel, I. C. Neue chronologische Angaben der Würm-Vereisung in Ungarn. *Földrajzi Közlemények* **18**, 5–18 (1969).

40. Konrád, G., Kovács, J., Halász, A., Sebe, K. & Pálffy, H. Late Quaternary woolly mammoth (Mammuthus primigenius Blum) remains from southern Transdanubia, Hungary. *Comptes Rendus - Palevol* **9**, 47–54 (2010).

41. Wilczyński, J. *et al.* Mammoth hunting strategies during the Late Gravettian in Central Europe as determined from case studies of Milovice I (Czech Republic) and Kraków Spadzista (Poland). *Quat. Sci. Rev.* **223**, (2019).

42. Wilczyński, J. *et al.* New Radiocarbon Dates for the Late Gravettian in Eastern Central Europe. *Radiocarbon* **62**, 243–259 (2020).

43. Vinogradov, A. P., Devirts, A. L., Dobkina, E. I. & Markova, N. G. Radiocarbon dating in the Vernadsky Institute V. *Radiocarbon* **10**, 454–464 (1968).

44. Vogel, J. C. & Waterbolk, H. T. Groningen Radiocarbon Dates VII. *Radiocarbon* **9**, 107–155 (1967).

45. Pazonyi, P. Palaeoecology and stratigraphy of Quaternary mammalian communities in the Carpath ian Basin (A Kárpát-medence kvarter emlősfauna közösségeinek paleoökológiai és rétegtani vizsgálata). (Eötvös Loránd University, 2006).

46. Béres, S. *et al.* Zöld Cave and the Late Epigravettian in Eastern Central Europe. *Quat. Int.* **587**–**588**, 158–171 (2021).

47. Vogel, J. C. & Waterbolk, H. T. Groningen radiocarbon dates V. *Radiocarbon* **6**, 349–369 (1964).

48. Hertelendi, E. Radiocarbon age of a bone sample from the upper paleolithic settlement near Jászfelsőszentgyörgy. *Tiscium* **8**, 61–62 (1993).

49. Szolyák, P. New Radiocarbon Data with Stratigraphical, Climatic and Archaeological Contexts to the Palaeolithic Assemblage of Herman Ottó Cave, Miskolc-Alsóhámor, Northeast Hungary. *Praehistoria* **9**–**10**, 213–224 (2009).

50. Vogel, J. C. & Waterbolk, H. T. Groningen radiocarbon dates X. *Radiocarbon* **14**, 6–110 (1972).

51. Stieber, J. Oberpleistozäne Vegetationsgeschichte Ungarns im Spiegel anthrakotomischer Ergebnisse (bis 1957) (A magyarországi felsőpleisztocén vegetáció-története az anthrakotómiai eredmények (1957-ig) tükrében). *Földtani Közlöny* **97**, (1967).

52. Rasmussen, S. O. *et al.* A stratigraphic framework for abrupt climatic changes during the Last Glacial period based on three synchronized Greenland ice-core records: Refining and extending the INTIMATE event stratigraphy. *Quat. Sci. Rev.* **106**, 14–28 (2014).

53. Giesecke, T., Ammann, B. & Brande, A. Palynological richness and evenness: Insights from the taxa accumulation curve. *Veg. Hist. Archaeobot.* **23**, 217–228 (2014).

54. Lindeløv, J. K. mcp: An R Package for Regression With Multiple Change Points. *J. Stat. Softw.* (2020) doi:10.31219/osf.io/fzqxv.
